# Silver Nanoparticle Arrays onto Glass Substrates Obtained by Solid-State Thermal Dewetting: A Morphological, Structural and Surface Chemical Study

**DOI:** 10.3390/nano12040617

**Published:** 2022-02-11

**Authors:** Juan Agustín Badán, Elena Navarrete-Astorga, Rodrigo Henríquez, Francisco Martín Jiménez, Daniel Ariosa, José Ramón Ramos-Barrado, Enrique A. Dalchiele

**Affiliations:** 1 Instituto de Física, Facultad de Ingeniería, Universidad de la República, Julio Herrera y Reissig 565, C.C. 30, Montevideo 11000, Uruguay; abadan@fing.edu.uy (J.A.B.); dariosa@fing.edu.uy (D.A.); 2 Laboratorio de Materiales y Superficies (Unidad Asociada al CSIC), Departamentos de Física Aplicada & Ing. Química, Universidad de Málaga, E29071 Málaga, Spain; enavarrete@uma.es (E.N.-A.); marjim@uma.es (F.M.J.); barrado@uma.es (J.R.R.-B.); 3 Instituto de Química, Facultad de Ciencias, Pontificia Universidad Católica de Valparaíso, Av. Brasil, Valparaíso 2950, Chile; rodrigo.henriquez@pucv.cl

**Keywords:** silver, nanoparticles, solid state dewetting, nanostructured layers, facets

## Abstract

Silver nanoparticles (NPs) on glass substrates were obtained by a solid-state thermal dewetting (SSD) process using vacuum-evaporated-silver precursor layers. An exhaustive investigation of the morphological, structural, and surface chemistry properties by systematically controlling the precursor film thickness, annealing temperature, and time was conducted. Thin silver films with thicknesses of 40 and 80 nm were deposited and annealed in air by applying a combined heat-up+constant temperature–time program. Temperatures from 300 to 500 °C and times from 0 to 50 min were assayed. SSD promoted the morphological modification of the films, leading to the Ag NPs having a discrete structure. The size, shape, surface density, and inter-nanoparticle distance of the nanoparticles depended on the initial film thickness, annealing temperature, and time, exhibiting a cubic silver structure with a (111) preferred crystallographic orientation. The prepared NPs were found to be highly enriched in the Ag{111}/Ag{110}/Ag{100} equilibrium facets. SSD not only promotes NP formation but also promotes the partial oxidation from Ag to AgO at the surface level. AgO was detected on the surface around the nanoparticles synthesized at 500 °C. Overall, a broad framework has been established that connects process factors to distinguish resultant Ag NP features in order to develop unique silver nanoparticles for specific applications.

## 1. Introduction

Noble metallic nanoparticles (Au, Pt, Ag) (NPs) have attracted a great deal of attention due to their unique physical features and interesting electrochemical and mechanical properties [1,2,3,4,5,6]. In particular, their optical properties, which have been known since antiquity, have already proven a fraction of their potential, and further major discoveries should be expected in the future [2,7,8,9]. In fact, one of the most important properties of such nanoparticles is the result of the interaction of these metallic nanostructures with incident light, generating collective oscillation modes of the conduction electrons in the particles, called plasmons, which become dominant and produce the surface plasmon resonance (SPR), resulting in the appearance of an electromagnetic field that is localized on the NPs [6,7,9,10,11,12]. The localized surface plasmon resonance (LSPR) phenomenon arises when the oscillations of the electromagnetic field of an incoming electromagnetic wave are in resonance with those of the local electromagnetic field of the NPs, which is defined by the resonance oscillation frequencies (both in the visible and near-infrared ranges., i.e., 400–1000 nm) [7,8,9,13]. More importantly, the resonant wavelengths of the LSPR can be tuned through the shape, size, uniformity, density, spacing, arrangement, type of the metallic nanoparticles, and their environment [2,6,7,9,10,13]. Furthermore, the physico-chemical characteristics of noble metallic NPs may be tailored extensively by manipulating their size and shape, which is a critical problem for a variety of technological applications [2,4]. LSPR also boosts Raman and fluorescence signals by creating large field enhancement [2,7]. As a result, noble metal nanoparticles have been rapidly produced for applications such as surface-enhanced Raman scattering (SERS) [14,15]. Due to their unique optical properties, the noble metallic nanoparticles detailed and discussed above have been intensively employed in a broad range of applications in the biomedical (bioimaging, biosensing, nanosurgery, drug delivery, photothermal therapy), energy, catalysis, and information technology (data storage) fields [2,7]. The development of single-molecule analytical systems has received a lot of interest in recent years; as a result, innovative sensing technologies based on localized surface plasmons in metallic nanoparticles have received a lot of attention (i.e., single-molecule sensing by fluorescence enhancement and plasmon shifts) [9,16].

Because of its strong resonance and low optical losses in the visible and near infrared parts of the spectrum, Ag, a cost-effective noble metal, has been frequently employed among many metallic NPs [2,17,18,19,20]. For instance, when designing plasmon-enhanced solar cells, Ag NPs are preferred over Au NPs because Ag NPs provide significantly larger path length increases [19,21]. As a result, Ag NPs are now employed in a variety of applications due to their distinctive physical, chemical, electrical, and size-dependent optical capabilities; Ag NPs have been used in the following applications: targeted drug delivery, wastewater treatment, biosensors, antimicrobial activity, solar cells, photo catalysis, and catalytic activity as well as for the detection of chemical species via SERS [2,5,10,20].

Thus, in the context of the huge developments that have been made in noble metallic nanoparticle application areas in recent years, several methods for silver nanoparticle synthesis have been described [5,22,23]. Chemical and electrochemical techniques, thermal breakdown, microwave synthesis, sonochemical synthesis, microemulsion synthesis, solvothermal synthesis, co-precipitation, and photoreduction have all been employed in the fabrication of Ag NPs [5,22,23,24]. Although most of these chemical and physical methods produce Ag NPs of good quality (desired shape and size), some of these methods synthesize these nanoparticles in a liquid phase, and the Ag NPs that are prepared remain in the form of colloidal solutions [5,10,22,23,24,25]. 

However, in many emerging nanoelectronic and optoelectronic technologies and applications, such as nanophotonic devices, new-generation solar cells, magnetic recording, sensors, and plasmonics, patterning metallic nanoparticles onto a substrate surface is desired [1,13,25,26,27,28,29], which, in the case of Ag NP colloidal solutions, the organization of the synthesized nanoparticles is difficult to control, and periodic arrays are challenging to realize [1,25,27]. In particular, the structure and properties of the metal nanostructures placed on nonmetal surfaces are of tremendous interest due to their potential use in different electronic, magnetic, and optical devices [30]. As a result, the development of simple, multipurpose, and low-cost methodologies for fabricating metallic nanoparticle arrays with the desired shape, size, and arrangement directly onto a solid substrate surface is the main critical issue for the use of metal nanostructures in order to make solid-state technological applications possible [4,28,31,32]. Moreover, and very importantly, in order to ensure the desired control of nanostructures, it is critical to have a thorough understanding of the fundamental microscopic mechanisms that govern the processes used in these approaches [4,28,31,32]. Several techniques have been reported for the production of micro- and nanoscale metallic particles arrays supported onto a solid substrate, including spray pyrolysis [33,34], sol–gel [35], electroless metal deposition [34], electrodeposition [34], and solid-state dewetting (SSD) [4,25,36,37,38,39,40].

Solid-state dewetting is a simple, low-cost, and industrially known scalable approach for the fabrication of substrate-bound metallic nanoparticles arrays onto various substrates, including glass, α-Fe_2_O_3_(0001)/α-Al_2_O_3_(0001), α-Fe_2_O_3_(0001)/SrTiO_3_(111), and ceramic single-crystals such as *c*-plane sapphire (α-Al_2_O_3_) and silicon [38,41,42,43,44]; in recent years, it has received a lot of interest as a viable approach for the large-scale fabrication of advanced photonic nanostructures [25,28,41,45,46,47]. SSD is a spontaneous phenomenon where a continuous polycrystalline metallic thin film onto a surface rearranges itself into an ensemble of separated islands or droplets upon appropriate annealing [25,28,41,45,46,47,48]. The original continuous film breaks apart into numerous particles in the same way as a water layer would on a hydrophobic surface, and this process is known as dewetting [25,28,41,45,46,47,48]. The SSD process can be induced by thermal annealing [12,26,31,36,38,40,41,49], pulsed laser heating [1,4,39,50], combined thermal annealing and pulsed laser heating [27], ion beam irradiation [51,52,53,54], electron beam irradiation [55], and inductively coupled plasma discharge [56]. Nonetheless, thermal annealing is probably the simplest way to promote dewetting, particularly when working on large areas. Deposited metallic films with thickness of a few nanometers are defective metastable layers that are comprised of polycrystalline grains (formed in a Volmer–Wever growth mode) with nanoscale islands that strain toward each other as they merge [25,28,41,46,47,57,58]. Then, dewetting can occur at temperatures considerably below the melting point of the material, highlighting the island structure, which appears in the initial pristine polycrystalline precursor film [25,28,41,46,47,57]. The main driving force for SSD is the reduction in the total free energy at the interfaces between the air, film, and substrate, which occurs via diffusive mass transfer at the surface, interface, or grain boundaries [25,28,41,45,46,47,48,59]. Thus, controlling the SSD process parameters in a precise way creates the possibility of obtaining nano-objects with a pre-defined statistical size and spatial distribution (starting from a thin polycrystalline metallic film), avoiding complicated and expensive lithographical subtractive processes [28,46].

In this work, silver nanoparticle arrays onto glass substrates are obtained by a solid-state thermal dewetting process using vacuum-evaporated polycrystalline silver precursor layers. An exhaustive investigation of the morphological, structural, and surface chemistry properties by means of the systematic control of the precursor film thickness, annealing temperature, and time is carried out.

## 2. Materials and Methods

### 2.1. Preparation of the Silver Nanoparticle Arrays onto Glass Substrates by Solid-State Dewetting

Plain soda lime glass microscope slide substrates that were 75 × 25 mm^2^ in size were used. First, they have were cut into smaller rectangles that were 2 cm^2^ in size. Further and prior to deposition, these glass substrates were submitted to ultrasonic cleaning with acetone, isopropanol, and water, with the samples being placed in each solution for 15 min. They were then dried with a dry air flux. 

After, a silver layer with a controlled thickness (nominal thickness, d_Ag_) was deposited onto these cleaned glass substrates by vacuum thermal evaporation at a high vacuum rate with ~10^−6^ Torr of base pressure (Intercorvamex, México City, México); Ag pellets with a purity of 99.99 % were used (Aldrich, St. Louis, MO, USA). The deposition rate was optimized at 1–2.5 Å/s to grow good-quality uniform films. The thicknesses of these films were monitored using an INFICON front load single sensor with a quartz crystal. This was connected to an INFICON STM-2 USB thin film rate/thickness monitor. The post-deposition annealing process to trigger solid-sate dewetting was performed in a quartz tube furnace at a temperature T_A_ for an annealing time t_A_. 

### 2.2. Morphological and Structural Characterization

Field-emission scanning electron microscopy (FE-SEM) pictures of the Ag NPs that formed on the glass substrate surface were obtained on a Helios Nanolab 650 Dual Beam from FEI equipment company. These FE-SEM images were taken under the following conditions: 2 kV and 100 pA for the operating voltage and current, respectively; in Mode II, immersion was determined through the lens detector.

The surface morphology of the deposits was examined using an atomic force microscope (AFM). Topographic AFM examinations were performed using a Nanoscope V tapping-mode AFM (Veeco Instruments, Plainview, NY, USA) by employing sharp silicon tips.

Transmission electron microscopy (TEM), high-resolution transmission electron microscopy (HRTEM), and selected-area electron diffraction (SAED) were carried out with a Philips CM-200 microscope operated at 200 kV to obtain the orientation of the crystalline plane, crystal size, and the distance between them.

The structural characterization of the sample was determined by X-ray diffraction (XRD) by using a PANalytical X’Pert PRO MRD diffractometer and a PANalytical PW3050/65 (Theta/2Theta) goniometer. X-ray source: sealed tube, ceramic Cu Anode. 

The FE-SEM images were studied using Image J software to obtain the size distributions, circularity, and autocorrelation functions of the Ag NPs on each sample. In the case of the AFM images, they were analyzed using Gwyddion software.

Circularity *C* was calculated using Image J software. Using ellipse fits, the area and perimeter of each NP were obtained. Subsequently *C* was calculated as the mean value of $\frac{4\pi *area}{perimeter^{2}}$.

### 2.3. Surface Chemistry Analysis

X-ray photoelectron spectroscopy XPS was carried out with a Versaprobe II equipment with a focused monochromatic Al Kα X-ray source (1486.6 eV) and a Physical Electronics PHI 5700XPS with Mg Kα (1253.6 eV) radiation, both of which were from Physical Electronics PHI, Chanhassen, MN, USA. The binding energies were corrected against to determine C at a 1 s peak of adventitious carbon fixed at 284.8 eV. The samples were analysed as obtained, and they were later etched with 4 KeV Ar^+^ ions for 1 min.

## 3. Results and Discussion

### 3.1. Synthesis of the Ag Nanoparticle Arrays onto Glass Substrates

**Figure 1:**
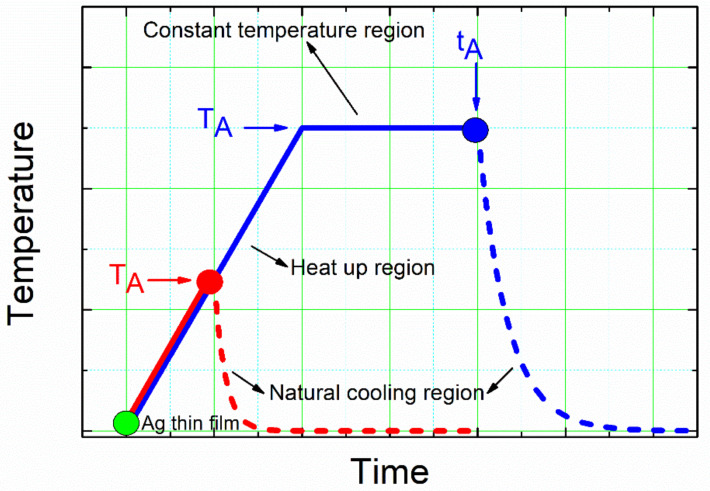
Schematic diagram of the temperature–time profile program employed for the annealing step of the solid-state dewetting process, where the different annealing and cooling to room temperature regions are indicated. Red and blue areas were used to determine the effects of the annealing temperature (T_A_) and the annealing time (t_A_), respectively, on the morphological and structural properties of the resulting silver nanoparticles/glass substrate samples.

Silver nanoparticle (NP) arrays were formed on the glass surface through the solid-state dewetting (SSD) process, which consists of two steps: (i) a precursor silver layer is deposited onto glass substrates, and (ii) these samples are then submitted to an annealing treatment to trigger the actual solid-state dewetting process. Starting film thickness, initial film crystallographic orientation, annealing temperature, annealing duration, and annealing environment are the key factors of the film and process that determine nanoparticle formation and the final morphology and size of the nanoparticles [28,47,60]. In the present case, all of the above-mentioned critical enumerated parameters: the thickness of the initial silver layer (d_Ag_), annealing temperature (T_A_), and the annealing time (t_A_), have been taken into consideration as variable parameters. Figure 1 depicts the temperature–time profile program of the annealing step of the solid-state dewetting process employed in present study. In an attempt to experimentally uncouple the effects of temperature and time on the SSD mechanism, this temperature–time profile program was employed. In the diagram below, two different zones can be identified: the heat-up region (temperature–time ramp) and the constant temperature region (temperature-time plateau). 

The first test allowed us to study increases in the annealing temperature and the evolution of the precursor silver thin films (for different d_Ag_ values) during the formation of the Ag NPs during this step of the SSD process (allowing for more accurate thermodynamic data). The second experiment allowed us to study the effect of the annealing time at different T_A_ values and for different d_Ag_ values during the formation of the Ag NPs during this step of the SSD process (allowing for more accurate kinetic data). Dewetting is usually noticed at a specific temperature, T_dewet_, which depends on the metal and on its thickness [47]. In the case of silver layers with thickness values of 20 nm to 80 nm, the T_dewett_ value is between 300 °C and 500 °C (well below its melting temperature T_m_ = 961 °C) [47,61]. Because of this, in present study, based both on this T_dewett_ value and the mechanical stability of glass substrates (whose transformation temperature is 525 °C), the maximum studied temperature was 500 °C. In both study cases (temperature-time ramp and temperature-time plateau), the natural cooling of the samples until they reached room temperature was applied as part of the experimental protocol. 

### 3.2. Morphological Characterization of the Ag Nanoparticle Arrays

**Figure 2:**
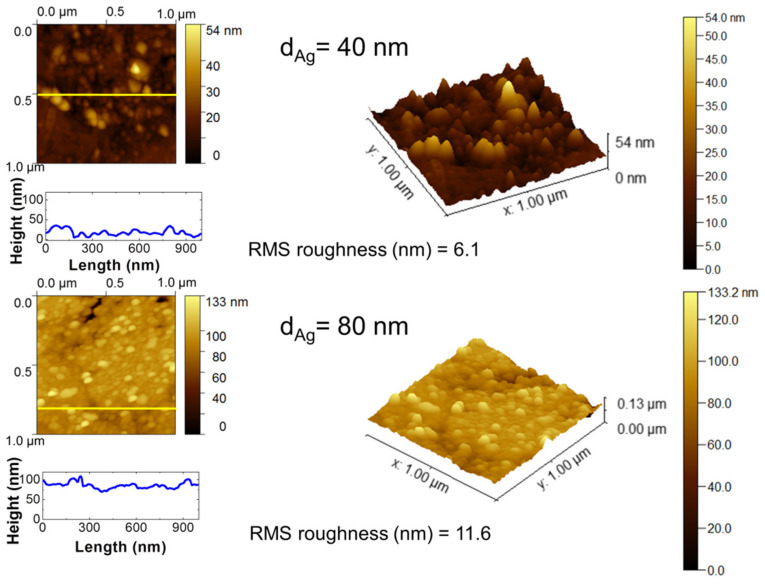
The 2D and 3D AFM topography scan images on 1 µm × 1 µm areas of vacuum-thermal-evaporated silver layers on glass substrates with nominal thicknesses of d_Ag_ = 40 nm and d_Ag_ = 80 nm, as indicated. The RMS roughness values are indicated. Cross-sectional surface line profiles obtained from the yellow lines of the corresponding AFM micrograph images are depicted.

As mentioned above, the first step in the SSD process is the deposition of a precursor silver layer onto the glass substrates via vacuum thermal evaporation. In the present work, two different silver layer thickness d_Ag_ were studied, i.e., d_Ag_ = 40 nm and 80 nm. Figure 2 shows representative 2D and 3D AFM images of these silver layers, which show the surface aspects and the morphological characteristics. AFM analysis was limited to smaller areas (1 µm × 1 µm) that were randomly chosen on the substrates to provide a better morphological characterization of the silver precursor layers. In the case of a silver layer with d_Ag_ = 40 nm, a rough dome-shaped nanostructured surface with a surface roughness value (RMS) of 6.1 nm can be seen, whereas for the thicker silver layers (d_Ag_ = 80 nm), a dome-shaped nanostructure with a rougher surface can be appreciated with a surface roughness value RMS = 11.6 nm, as shown by the cross-sectional line profiles in Figure 2. The last step of the Volmer–Weber growth process for metal thin films on a glass substrate is characterized by these rough granular morphologies [31]. Figure 2 shows that in the case of a silver layer with d_Ag_ = 40 nm, the silver nanoparticles exhibited a mean size of 42 nm, and some isolated areas with higher height values with respect to the whole inspected surface are depicted; whereas for the thicker layers (d_Ag_ = 80 nm), the silver nanoparticles with a mean size of 56 nm are dispersed and have a more homogeneous height distribution. 

**Figure 3:**
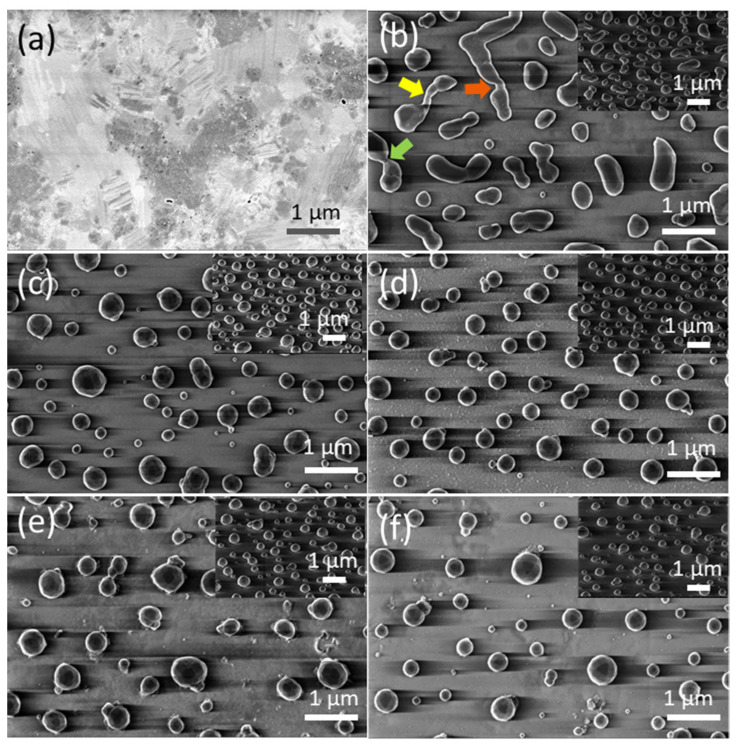
Top views of the FE-SEM micrographs of vacuum-thermal-evaporated silver layers with a nominal thickness d_Ag_ = 40 nm at different stages of the annealing step during the SSD process applying the heat-up region program depicted in Figure 1 and ending at different TA values: (**a**) as-grown silver layer, (**b**) T_A_ = 250 °C, (**c**) T_A_ = 300 °C, (**d**) T_A_ = 350 °C, (**e**) T_A_ = 400 °C, and (**f**) T_A_ = 450 °C. The insets depict the corresponding tilted FE-SEM micrograph views (with a tilt angle of 52°). The colored arrows in (**a**) highlight specific features of the dewetting process, leading to the formation of silver nanoparticles. Temperature ramp rate =10 °C /min. In all of these cases, a soda lime glass substrate has been used.

**Figure 4:**
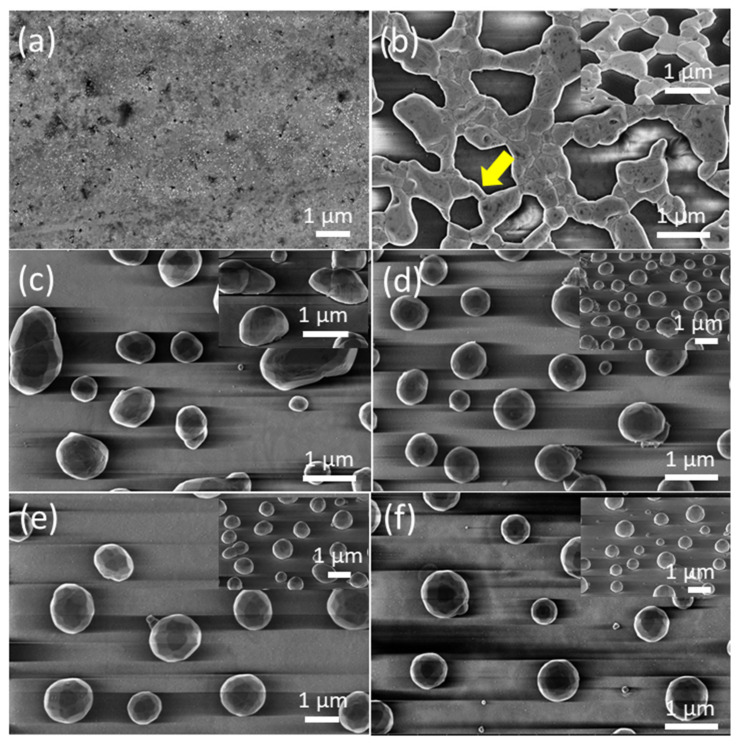
Top views of the FE-SEM micrographs of vacuum-thermal-evaporated silver layers with a nominal thickness d_Ag_ =80 nm at different stages of the annealing step during the SSD process applying the heat-up region program depicted in Figure 1 and ending at different T_A_ values: (**a**) as-grown silver layer, (**b**) T_A_ = 250 °C, (**c**) T_A_ = 300 °C, (**d**) T_A_ = 350 °C, (**e**) T_A_ =400 °C, and (**f**) T_A_ = 500 °C. The insets depict the corresponding tilted FE-SEM micrograph views (with a tilt angle of 52°). The yellow arrow in (**b**) highlights specific features of the dewetting process, leading to the formation of the silver nanoparticles. Temperature ramp rate =10 °C /min. In all cases, a soda lime glass substrate has been used.

Figure 3a and Figure 4a show FE-SEM micrographs of as-grown precursor silver thin films with thicknesses of d_Ag_ = 40 nm and d_Ag_ = 80 nm, respectively, deposited on a glass substrate via vacuum thermal evaporation. Well-covered and continuous layers can be observed. Figure 3b–f and Figure 4b–f show FE-SEM micrograph images of vacuum-thermal-evaporated silver layers with nominal thicknesses d_Ag_ = 40 nm and d_Ag_ = 80 nm, respectively, at different stages of the annealing step during the SSD process applying the heat-up region program depicted in Figure 1 and ending at different T_A_ values, as indicated; this demonstrates that the silver thin films undergo a transition from continuous to discontinuous as exposure to the glass substrate increases. At a first glance, the FE-SEM micrographs illustrate that annealing at lower temperatures produces larger, elongated silver particles than annealing at higher temperatures, which produces smaller, more spherical silver particles. 

In the low annealing temperature regime, which takes place at temperatures up to T_A_ = 250 °C, the formation of irregular and elongated clusters is observed in the d_Ag_ = 40 nm (Figure 3b) samples, and in d_Ag_ = 80 nm samples, the presence of coalesced nanostructures and a high percolation degree is noticeable (Figure 4b). Figure 4b also shows the first stages of hole formation. As the T_A_ increases from 300 °C to 450 °C (in the case of samples with d_Ag_ = 40 nm) or 500 °C (in the case of samples with d_Ag_ = 80 nm), those previously observed structures lead to well-separated islands that agglomerate into elongated and oval nanoparticles, as seen Figure 3c–e and Figure 4c–e, and the oval-like nanoparticles will shrink into spherical nanoparticles with a relatively uniform size at elevated temperatures (Figure 3f and Figure 4f) due to surface tension. In particular, in Figure 3b, the colored arrows indicate specific features occurring during the annealing step of the SSD process.

The hole coalescence can be recognized in different situations, i.e., two particles connected by a residual filament alone (indicated by the yellow arrow); two big particles connected by a neck (indicated by the dark orange arrow) and two filaments that are in contact and about to separate (indicated by the light green arrow). Similarly, in Figure 4b, it can be seen two big grains are still attached by an isthmus, which is indicated by a yellow arrow. The further evolution of the dewetting process (in this case by increasing T_A_) leads to the shrinking of the diameter of the filaments until complete filament rupture occurs and round silver nanoparticles are formed.

**Figure 5:**
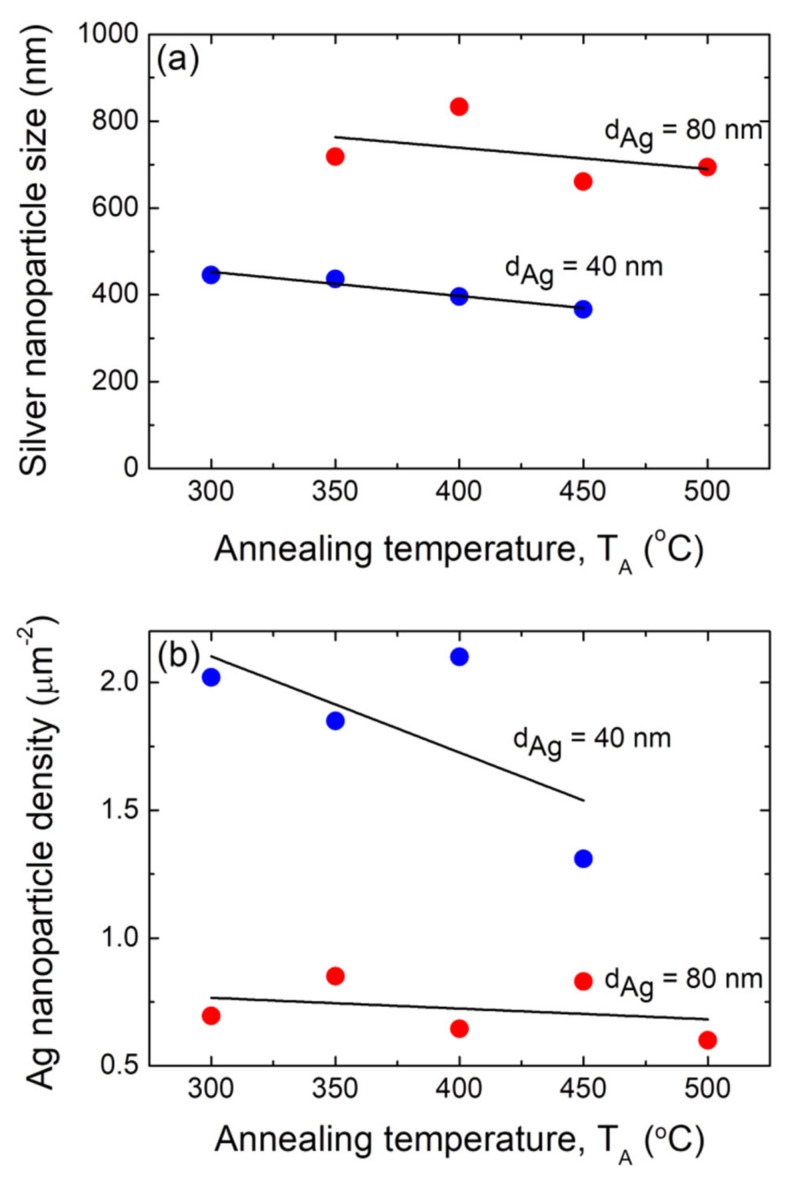
(**a**) Plot of the average size of the silver nanoparticles as a function of the annealing temperature T_A_ of the annealing step during the SSD process. (**b**) Plot of surface density of the silver nanoparticles as a function of the annealing temperature T_A_ during the annealing step of the SSD process. Two different precursor silver layer thickness were studied, i.e., d_Ag_ = 40 nm (blue circles) and 80 nm (red circles). In all of these samples, the heat-up region program previously depicted has been applied. The black lines are a guide for the eyes.

The size distribution histograms corresponding to the morphological quantitative study of those silver nanoparticle arrays depicted in Figure 3 and Figure 4 are presented in Figures S1 and S2, respectively. Providing the basis for these results, Figure 5a shows plots of the mean size (D) of the formed Ag NP arrays as a function of the annealing temperature T_A_ for silver precursor layer samples with the two different thickness, i.e., d_Ag_ = 40 nm and 80 nm. It can be appreciated that the Ag NPs that were formed from a precursor layer with a thickness of 80 nm exhibited greater mean sizes than those obtained from a silver layer with a thickness of 40 nm, irrespective of the annealing temperature. As mentioned above, one of the critical parameters controlling the final morphology of the silver NPs is thin film thickness, d_Ag_. The initial film thickness is the main parameter that determines D, whereas the annealing temperature is responsible for obtaining round nanoparticles [62]. The increase in Ag NP size as the precursor for the increases in the film thickness is consistent with what has been stated and documented in the literature [60,62,63,64]. In fact, in the SSD process, D is proportional to d_Ag_, according to the theoretical basis described elsewhere, and this will be presented and discussed later in this work [47,60]. Moreover, it can be seen that for both precursor layer thicknesses (40 and 80 nm), the mean size of the Ag NPs decrease slightly as the annealing temperature increases (see Figure 5a). This behavior is in line with that reported by Serrano et al. for SSD-dewetted silver NPs formed on soda lime glass substrates (from Ag films with similar thicknesses, 30 nm and 50 nm) and by applying a similar annealing temperature range as the one in the present work; however, a different annealing temperature–time profile was used [38]. However, Serrano et al. reported a decrease in the Ag NP size of about 2 nm per degree Celsius, and in our case, a slight nanoparticle size decrease of about 0.5 nm per degree Celsius was observed. Oliva-Ramirez et al. also reported that the particle size decreases as the temperature increases during the SSD-induced formation of AuNi alloy nanoparticles [65].

Figure 5b shows plots of the silver nanoparticle surface density (N) as a function of the annealing temperature T_A_ for samples with two different silver precursor layer thickness, i.e., d_Ag_ = 40 nm and 80 nm. It can be seen that the surface density of the Ag NPs formed from a precursor layer with a thickness of of d_Ag_ = 40 nm exhibited greater N values than those obtained from a silver layer with a thickness of of d_Ag_ = 80 nm, irrespective of the annealing temperature. As reported in the literature, variations in the NP density d_Ag_^−2^ are expected [60], and the results presented in Figure 5b are in agreement with these statements. Furthermore, it can be seen that for both precursor layer thicknesses, the surface density of the Ag NPs decrease slightly as the annealing temperature T_A_ increases (see Figure 5b). Serrano et el. [38] observed that as the covered area decreases, the island height and catchment area increases when the annealing temperature is higher. This can be associated with a decrease in the density.

**Figure 6:**
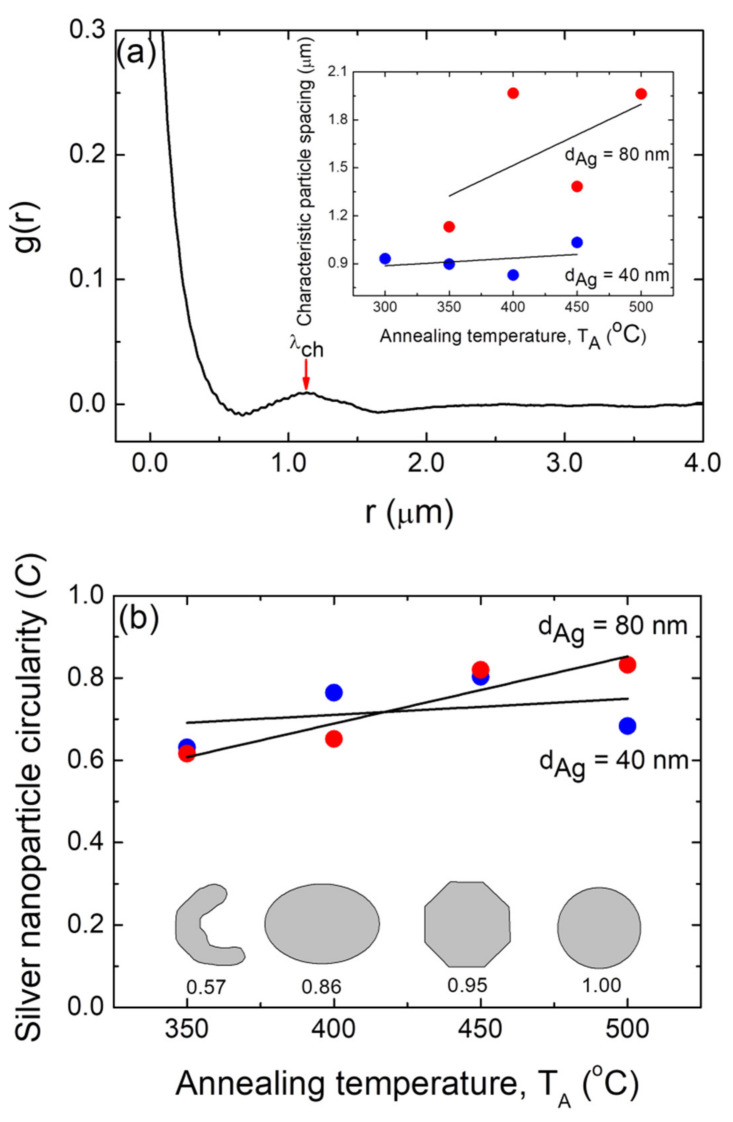
(**a**) Typical measured radially averaged autocorrelation function, g(r), for the silver nanoparticle positions for a typical sample with d_Ag_ = 80 nm and T_A_ = 350 °C. Arrow indicates the corresponding characteristic particle spacing λ_ch_ (at maximum). Inset: positional correlation as a function of the annealing temperature, T_A_, for samples with two different thicknesses: d_Ag_ = 40 nm (blue circles) and 80 nm (red circles). (**b**) Mean circularity of the silver nanoparticles formed as a function of the annealing temperature, TA, for samples with two different thicknesses: d_Ag_ = 40 nm (blue circles) and 80 nm (red circles). Inset: elongated, oval, polyhedral, and circular shapes and their corresponding circularities. In all of these figures, the black lines are guides for the eyes.

The organization of the supported metallic nanoparticles is required for nanotechnology applications [55,66]. To quantify the ordering, we calculated the experimental radially averaged autocorrelation function (g(r)) using FE-SEM micrograph images [36,37,55,65,67]. The autocorrelation of the pixels in a binary micrograph image as a function of their radial distance (also known as pair correlation) was used to determine the radially averaged autocorrelation. As an example of this procedure, the pair correlation function plot for the silver nanoparticles generated from an 80 nm thick Ag layer annealed at T_A_ = 350 °C is shown in Figure 6a, which corresponds to an FE-SEM micrograph image from Figure 4c (see Figures S3 and S4 for plots of the g(r) functions for the silver nanoparticle array samples formed under the other studied conditions of d_Ag_ and T_A_). The first maximum indicates the characteristic length (particle spacing), λ_ch_. The g(r) function plot depicted in Figure 6a exhibits a peak at about 1.1 µm, which indicates that the silver NPs have a positional correlation, and the characteristic particle spacing λ_ch_ of the positional correlation is about 1.1 µm. The obtained characteristic particle spacing for each sample is plotted as a function of the annealing temperature, T_A_, for the two different silver layer thicknesses (d_Ag_ = 40 nm and 80 nm, as indicated) as an inset of Figure 6a. It can be appreciated that the Ag NPs formed from a precursor layer with a thickness of 80 nm exhibited greater characteristic particle spacing values than those obtained from a silver layer with a thickness of of 40 nm, irrespective of the annealing temperature.

The increase in the λ_ch_ values as the precursor for the increases in the thin film thickness is consistent with what has been said and documented in the literature for gold nanoparticles formed through the SSD process [55]. Moreover, as depicted in the inset of Figure 6a, the Ag NP arrays formed from a precursor layer with a thickness of 40 nm exhibited almost constant λ_ch_ values as a function of T_A_. On the other hand, those Ag NP array samples that were obtained from a precursor layer with d_Ag_ = 80 nm exhibited an increase in the λ_ch_ values as a function of T_A_ (see inset of Figure 6a), which is in agreement with previously reported results [36]. In fact, as discussed above, as the annealing temperature increases, smaller and less surface dense Ag NPs are obtained, resulting in the averaged inter-particle distance increasing, which can be seen in the inset of Figure 6a.

To further quantify those observations about the shape of the Ag NPs, Figure 6b presents their mean circularity *C* as a function of the annealing temperature T_A_ for samples with silver precursor layers with the two thicknesses used in this study, i.e., d_Ag_ = 40 nm and 80 nm. This parameter *C* is a shape descriptor (in-plane projected shape) that compares the actual area of the nanoparticles to that of a virtual circle of the radius *r* determined by the perimeter *P* (*r* = *P* ∕ 2*π*) [65]. Highly circular silver nanoparticles have the desired shape condition for the excitation of the narrower full-width half maxima of the localized surface plasmon resonance surface enhanced by spectroscopy as well as for sensors [68]. As can be appreciated in Figure 6b, by definition, a perfectly circular particle has a circularity of 1, whereas elongated, oval, or faceted particles have a circularity of less than one [65]. Figure 6b illustrates that at lower temperatures, Ag NPs are more elongated, and as the temperature rises, they become rounder. However, for the d_Ag_ = 40 nm case, lower *C* values and a less pronounced effect with T_A_ compared to those of the d_Ag_ = 80 nm samples can be appreciated.

**Figure 7:**
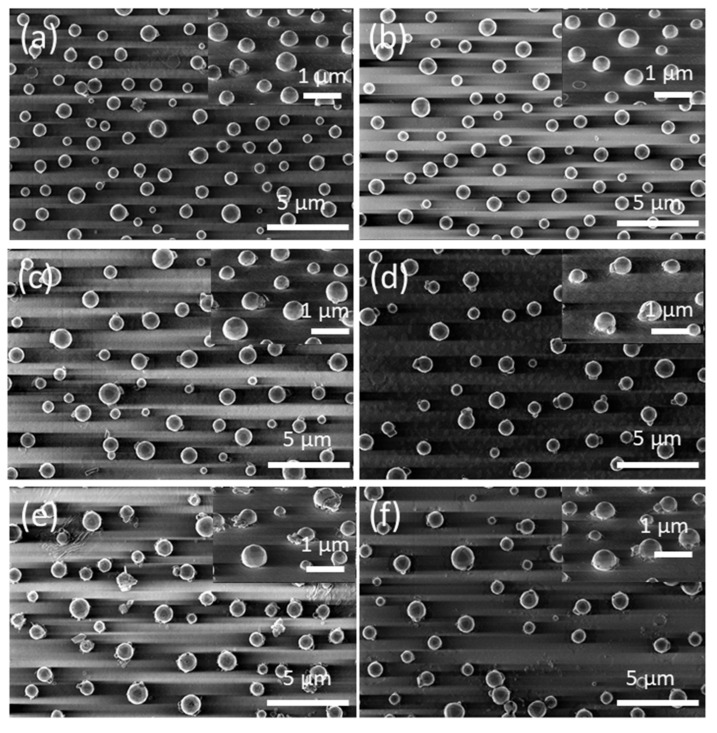
Top views of FE-SEM micrographs of vacuum-thermal-evaporated silver layers with a nominal thickness d_Ag_ = 80 nm at different stages of the annealing step during the SSD process applying the constant temperature region program depicted in Figure 1 at T_A_ = 500 °C and ending at different t_A_ values: (**a**) t_A_ = 8 min, (**b**) t_A_ = 17 min, (**c**) t_A_ = 26 min, (**d**) t_A_ = 34 min, (**e**) t_A_ = 42 min, and (**f**) t_A_ = 51 min. The insets depict the corresponding tilted FE-SEM micrograph views (with a tilt angle of 52°). Temperature ramp rate =10 °C /min. In all of these, cases a soda lime glass substrate has been used.

To examine the kinetic evolution of the precursor silver layers in greater depth, i.e., to study the effect of the annealing time t_A_ on the morphological properties of the formed silver nanoparticles, the constant temperature region (temperature-time plateau) depicted in Figure 1 was applied to the precursor silver layers. Figure 7 shows FE-SEM micrograph images of vacuum-thermal-evaporated silver layers with a nominal thickness d_Ag_ = 80 nm at different stages of the annealing step during the SSD process (by applying the temperature-time plateau region program depicted in Figure 1), with T_A_ = 500 °C and ending at different t_A_ values, as indicated (see Figure S5 for a similar study with d_Ag_ = 40 nm and T_A_ = 450 °C). It can be appreciated that for t_A_ = 8 min (see Figure 7a) and despite the short annealing time, only well-separated hemispherical silver nanoparticles can be seen (without the presence of percolated nanostructures), which is in agreement to particles depicted in Figure 4f, indicating the main role of T_A_ over t_A_.

**Figure 8:**
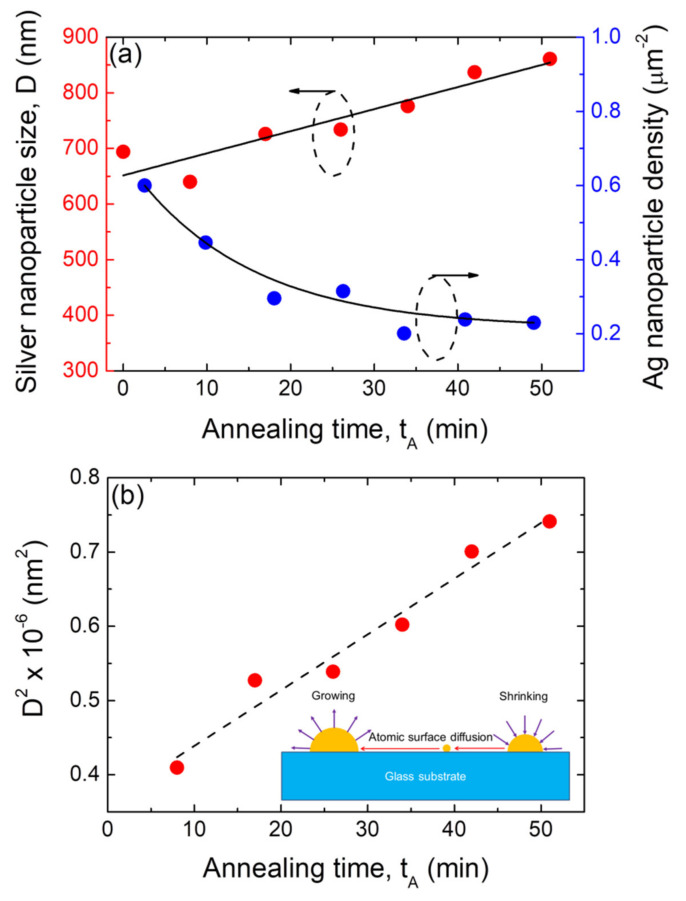
(**a**) Plots of the average size of the nanoparticles (D) and silver nanoparticle surface density as a function of the annealing time t_A_ of the annealing step of the SSD process. (**b**) Plot of the average size silver nanoparticles (D), where the square is a function of the annealing time t_A_ of the annealing step during the SSD process. The dashed black line corresponds to the linear fitting. SSD process for silver vacuum-thermal-evaporated layers with a nominal thickness of d_Ag_ = 80 nm applying the constant temperature region program depicted in Figure 1 at T_A_ = 500 °C. The solid black lines are guides for the eyes. Inset: Schematic to illustrate the diffusion of Ag ad-atoms and Ostwald’s Ag ripening during annealing.

Moreover, as the annealing time increased, the mean size of the hemispherical silver nanoparticles increase D. The size distribution histograms corresponding to the morphological quantitative study of those silver nanoparticle arrays depicted in Figure 7 are presented in Figure S6. As the basis of these results, Figure 8a shows plots of the mean size (D) and surface density (N) of the formed Ag NP arrays as a function of the annealing time t_A_ at the annealing temperature of 500 °C. It can be seen that as the annealing time increases, the mean size of the silver nanoparticles increases, whereas their surface density decreases.

The overall evolution of silver nanoparticle morphology, size, and density can be discussed based on the interplay between temperature-induced surface diffusion, the sublimation of silver atoms as well the surface energy minimization mechanism [49]. In fact, after the formation of the Ag NPs through the SSD mechanism, the concurrent impacts of surface diffusion, Ostwald’s ripening, and Ag sublimination can be used to explain the evolution of the morphology of these silver nanoparticles, which will be further addressed [6,10,30]. The temperature-dependent surface diffusion coefficient (*D_s_*) can be expressed by the following relationship [6,10]:

(1) $$D_{s}\propto e^{-\frac{E_{Ag}}{kT_{A}}}$$

where *T_A_* is the annealing temperature, *K* is the Boltzmann’s constant, and *E_Ag_* is the activation energy for diffusion of Ag ad-atoms. It is obvious that at a higher annealing temperature *T_A_*, the diffusion length (i.e., ad-atom displacement) can be larger, but at a lower temperature, it may be limited. On the other hand, high activation energy values lead to lower ad-atoms diffusion lengths.

Simultaneously, the metallic nanostructure evolution associated with the longer annealing duration at a fixed diffusion length with a constant annealing temperature can be described by the Ostwald’s ripening [6,10,30]. In the case of Ostwald’s ripening, as larger particles are more energetically stable than smaller ones, a driven spontaneous process occurs in which the larger NPs grow in size at the expense of the smaller ones as the annealing duration increases, as shown by the scheme in the inset of Figure 8b [6,10,30,69]. The difference in the chemical potential of atoms in nanoparticles of various sizes is the main driving factor for ripening [3]. Generally, the size of metallic NPs increases with the longer annealing duration based on Ostwald’s ripening, as previously discussed [6,10,30,69].

As described above, one of the dominant kinetic pathways for nanoparticle coarsening during the SSD process is Ostwald ripening (OR). OR kinetics can be divided into two extreme regimes: (a) terrace-diffusion-limited transport (with no significant barrier for attachment of diffusing species to cluster edges) and (b) attachment–detachment-limited transport (with a large attachment barrier) [30,70]. The last regime demonstrates mean field behavior, in which the average cluster size determines whether the cluster grows or decays [30,70], and in this regime case, the average nanoparticle radius *R_av_* scales with time (*t*) as follows [70]:

(2) Rav ∝ t12

**Figure 9:**
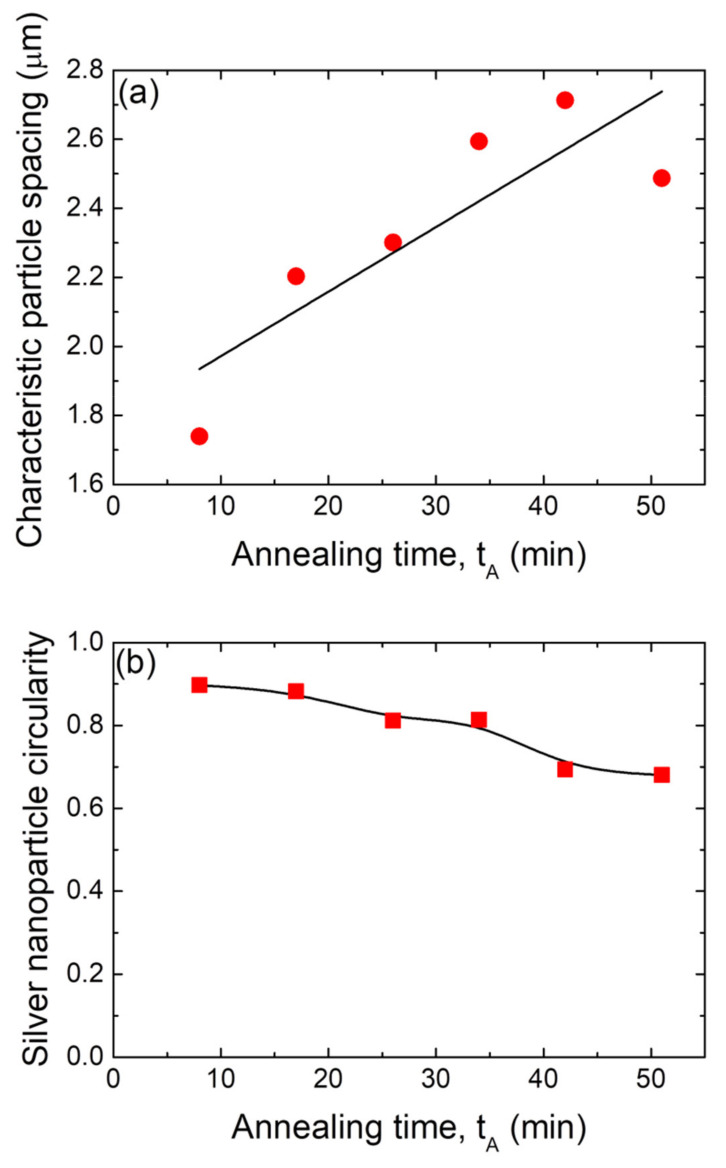
(**a**) Positional correlation as a function of the annealing time, t_A_, for samples with d_Ag_ = 80 nm and annealed at T_A_ = 500 °C. (**b**) Mean circularity of the silver nanoparticles formed as a function of the annealing time, t_A_, for samples with d_Ag_ = 80 nm and annealed at T_A_ = 500 °C. In all of these figures, the black lines are guides for the eyes.

As such, by plotting the square of the formed silver nanoparticle size vs. time, linear behavior is expected. In fact, Figure 8b shows a plot of the square of the experimental data for the silver nanoparticle size D^2^ as a function of the annealing time t_A_, which displays a linear relationship with good linear fitting (r = 0.982). This confirms the validity of Ostwald ripening under the conditions of attachment–detachment-limited transport regime for the growth of Ag NPs on glass substrates by means of the SSD process. Similar behavior was observed for the silver NPs that were formed from a precursor layer with a thickness of 40 nm and annealed at T_A_ = 450 °C at different times (see Figure S7). On the other hand, Figure 8a shows that as a consequence of the size growth of the Ag NPs as the annealing time increases, the Ag NP surface density decreases (based on mass conservation, bigger NPs have been formed but with lower quantity of them per unit area). The latter is also explained in Figure 9a, i.e., the increase in characteristic particle spacing with increased the annealing times.

Figure 9b presents the mean circularity *C* of silver nanoparticles (obtained from a precursor silver layer with d_Ag_ = 80 nm and annealed at T_A_ = 500 °C), as a function of the SSD annealing time. The Ag NP circularity with *C* = 0.9 remains nearly constant up to 20 min, but for greater times, it slowly decreases in steps to 0.7 at t_A_ = 51 min. As seen above (see Figure 8a), at longer times, larger Ag NPs are attained, indicating that this growth stage is also witness to a slight decrease in circularity, which means that at different stages of growth, larger particles have more asymmetry in their shape [68].

**Figure 10:**
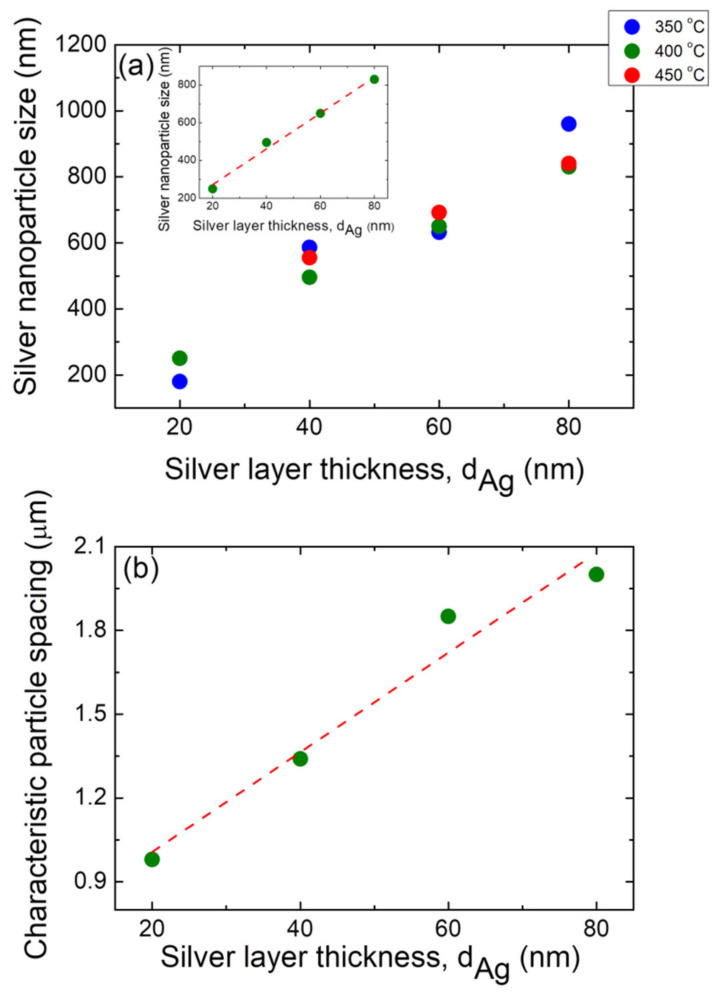
(**a**) Plot average size of the silver nanoparticles (D) as a function of the initial silver layer thickness, d_Ag_, for three different annealing temperatures: T_A_ = 350 °C (blue circles), T_A_ = 400 °C (olive green circles), and T_A_ = 450 °C (red circles). In all cases, an annealing time of t_A_ = 1 h has been applied. Inset: plot detail for the case of the T_A_ = 400 °C case. The dashed red line corresponds to the linear fitting. (**b**) Positional correlation as a function of the initial silver layer thickness, d_Ag_, for samples annealed at T_A_ = 400 °C for 1 h. The dashed red line corresponds to the linear fitting.

A study of the effect of the initial precursor silver layer thickness on the final formed Ag NP morphology was conducted. As example, FE-SEM micrograph images of vacuum-thermal-evaporated silver layers with four nominal thickness values: d_Ag_ = 20 nm, 40 nm, 60 nm, and 80 nm, and annealed at T_A_ = 400 °C (by applying the temperature-time plateau region program depicted in Figure 1) with t_A_ = 1 h, as indicated, are depicted in Figure S8. Figure 10a shows an increase in the mean size of the formed Ag NPs as a function of the thickness of the initial silver layer for samples annealed at three different temperatures: T_A_ = 350 °C, 400 °C, and 450 °C, as indicated, for t_A_ = 1 h. 

In fact, Jiran et al. [71] showed that the void growth rate *R_v_* decreases as the film thickness *d* dramatically increases, i.e., *R_v_* ∝ *d*^−3^; tit is then reasonable that both the nanoparticle size and the characteristic particle spacing (vide infra) increases with the precursor film thickness [37]. A linear increase in the mean Ag NP size with the precursor film thickness is also observed, regardless of the annealing temperature. In order to prove this linear behavior, an example is provided in the inset of Figure 10a, which shows a similar *D* vs. d_Ag_ plot, but only for the T_A_ = 400 °C, and also shows a linear fitting while exhibiting a good Pearson correlation coefficient (r = 0.995). This is in agreement with the findings reported in the literature for different layer materials and substrates [28,60,72]. Indeed, a similar linear dependence behavior for dewetted silver and gold films was observed by Morawiec et al. [62] and Kojima et al. [55], respectively. This shows that the initial film thickness is the most important factor in determining the mean NP size, whereas the annealing temperature determines the nanoparticle sphericity [62]. Figure 10b shows a linear increase in the characteristic particle spacing with the increase in the silver precursor film for a typical sample (T_A_ = 400 °C and t_A_ = 1 h). 

In this regard, Wang et al. [37], reported a non-linear behavior of λ_ch_ vs. the precursor film thickness in the preparation of gold nanoparticles during SSD process, whereas Kojima et al. [55] reported that Au NPS created with SSD a showed a linear behavior of λ_ch_ vs. the initial precursor film thickness, which was also observed in the present study.

### 3.3. Structural Characterization of the Ag Nanoparticle Arrays

**Figure 11:**
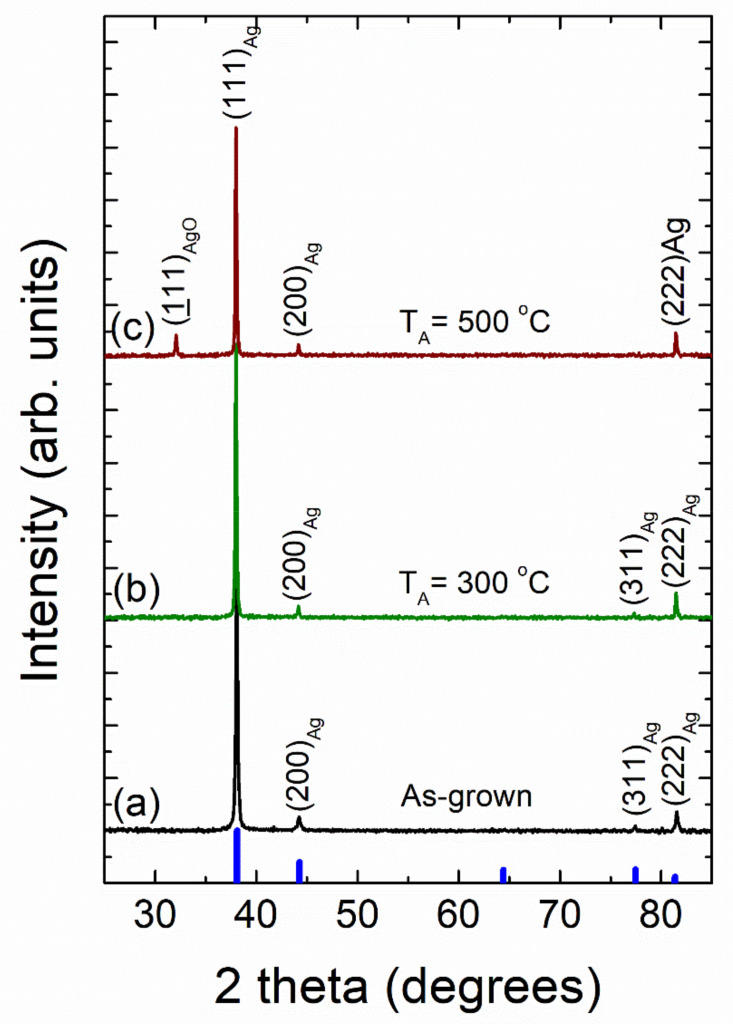
X-ray diffraction patterns of vacuum-thermal-evaporated silver layers with a nominal thickness d_Ag_ = 80 nm at different stages of the annealing step during the SSD process by applying the heat-up region program depicted in Figure 1 and ending at different T_A_ values: (**a**) as-grown silver layer, (**b**) T_A_ = 300 °C, and (**c**) T_A_ = 500 °C. Miller indices *(hkl)* for cubic silver diffraction planes are indicated. Cubic Ag JCPDS pattern is also shown for comparison (thick blue bars). Temperature ramp rate =10 °C /min. In all of these cases, a soda lime glass substrate has been used.

Figure 11 shows typical X-ray diffraction patterns of as-grown vacuum-thermal-evaporated silver layers with a nominal thickness d_Ag_ = 80 nm at different stages of the annealing step during the SSD process (applying the heat-up region program depicted in Figure 1) and ending at selected different T_A_ values, as indicated. At first glance, all the diffraction peaks can be indexed to the cubic silver structure (JCPDS file No. 04-0783) [73]. Four diffraction peaks corresponding to the (111), (200), (311), and (222) crystallographic planes of the cubic silver structure can be clearly identified in the as-grown and the solid-state dewetted samples, except for the sample annealed at T_A_ = 500 °C, which only exhibits the peaks at (111), (200), (222). 

**Figure 12:**
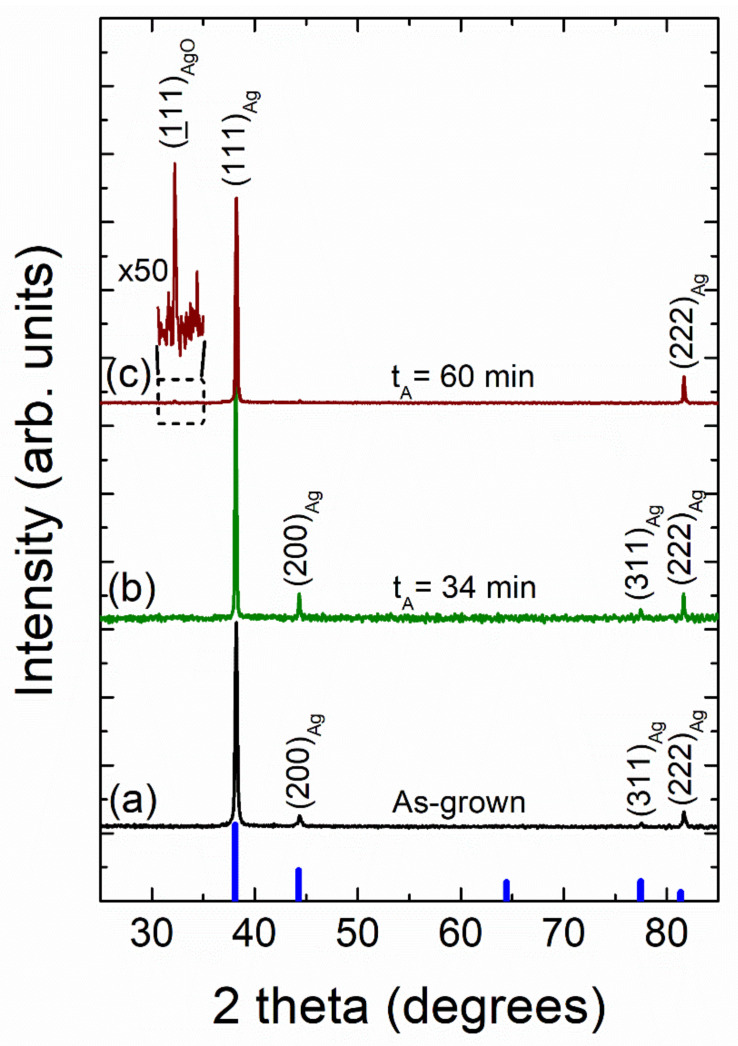
X-ray diffraction patterns of vacuum-thermal-evaporated silver layers with a nominal thickness d_Ag_ = 80 nm at different stages of the annealing step of the SSD process by applying the constant temperature region program depicted in Figure 1 at T_A_ = 500 °C and ending at different t_A_ values: (**a**) as-grown silver layer, (**b**) t_A_ = 34 min, and (**c**) t_A_ = 60 min. Miller indices (*hkl*) for cubic silver diffraction planes are indicated. Cubic Ag JCPDS pattern is also shown for comparison (thick blue bars). The zoom of the pattern (**c**) in the 2 theta region from 30 to 35 degrees depicts a diffraction peak corresponding to the AgO phase. Temperature ramp rate =10 °C /min. In all of these cases, a soda lime glass substrate has been used.

Figure 12 shows typical X-ray diffraction patterns of as-grown and vacuum-thermal-evaporated silver layers with a nominal thickness d_Ag_ = 80 nm at different stages of the annealing step during the SSD process (applying the temperature–time plateau region program depicted in Figure 1) with T_A_ = 500 °C and ending at different t_A_ values, as indicated.

Four diffraction peaks corresponding to the (111), (200), (311), and (222) crystallographic planes of the cubic silver structure can be clearly identified in the as-grown and the solid-state dewetted samples, except for the sample annealed during 60 min, which only exhibits the peaks at (111) and (222). In all of the studied cases, the large (111) Ag diffraction peak height (larger than the one expected for a random polycrystalline sample) indicates a strong crystallographic orientation ((111) out-of-plane texture and random in-plane grain orientations). Moreover, the other silver diffraction peaks all present a very low intensity. As a result, both the as-grown film and the silver nanoparticle array samples appear to have significant texturing. In order to quantify this fact, the texture coefficient TC(*hkl*) was calculated (see SI) [74,75,76,77]. The results of the texture analysis of the as-grown silver layers provides a value of TC(*111*) = 2.78 for a maximum of 3.0, indicating that the silver crystallites have a strongly preferred orientation along the <111> crystallographic direction. In general, for non-epitaxial thin film deposition on a substrate, the film surface tends to be crystallographically orientated at either (*111*) or (*001*) due to the minimum surface free energy exhibited by these planes [78]. Silver layer samples (with a nominal thickness d_Ag_ = 80 nm), which were submitted to the SSD process at different temperatures T_A_ (see XRD patterns in Figure 11), exhibited TC(*111*) values of approximately 2.8, which are nearly constant independent of the annealing temperature. On the other hand, the texture results on similar silver layer samples submitted at different stages of the annealing step of the SSD process (by applying a T_A_ = 500 °C and ending at different t_A_ values, see XRD patterns in Figure 12) showed a very slight increase in the TC(*111*) value as the t_A_ increased, reaching a TC(*111*) = 3.0 at t_A_ = 60 min. As such, as the preferred crystallographic orientation of the studied samples do not show a significant change after the SSD process; it can be inferred that the texturing and of the Ag NPs seems to be determined by the starting silver precursor layer. In fact, as previously observed, dewetted nanoparticle crystallographic alignments are linked to the formation of low-energy facets along specific crystallographic directions, which are substantially influenced by the initial film orientation [28]. In addition, as can be seen in Figure 11 and Figure 12, the silver (111) diffraction peak presents a very sharp profile, indicating that both the as-grown silver layer and the Ag nanoparticles are highly crystalline (high crystallite size values) and have a cubic structure. Crystallite size generally corresponds to the coherent volume in the material for the respective diffraction peak [79,80]. An estimation of the size of the crystallite coherent scattering domain (D_C_) was derived from the Scherrer equation (see SI) and for all cases calculated from the (111) diffraction peak. The as-grown silver films exhibited a D_C_ = 50 nm. Moreover, pristine silver layers submitted to the SSD process either by applying the heat-up region (at different temperatures T_A_) see XRD patterns in Figure 11) or by applying the constant temperature region (T_A_ = 500 °C and ending at different t_A_ values), see XRD patterns in Figure 12) exhibited D_C_ values higher than those of the as-grown ones. The last indication that the SSD process not only triggers the formation of separate silver nanoparticles, but it does so in a concomitant way that increases the sizes of the crystallites. It has been observed that the D_C_ increases from 70 to 90 nm as the T_A_ increases from 300 to 500 °C, and it remains nearly constant at D_C_ = 90 nm with t_A_ (0 to 80 min, @ T_A_ = 450 °C), showing that the annealing temperature has a greater effect on the crystallite size than the annealing time.

Furthermore, the XRD patterns of the samples annealed at T_A_ = 500 °C and at T_A_ = 500 °C for 60 min (by applying in each case the heat-up and temperature–time plateau region programs depicted in Figure 1, respectively) exhibited the presence of a diffraction peak at 2θ ~ 32.3° that can be assigned to a monoclinic silver oxide (AgO) phase (JCPDS file No. 74-1743 [81]) (see Figure 11c and Figure 12c, respectively). In fact, it has been reported that the silver oxide layer covering Ag NPs is formed at surface level during the annealing process, and as expected from a thermodynamic and kinetic point of view, the higher the annealing temperature T_A_, and the longer the annealing time, t_A_, the higher the degree of oxidation in the samples [38,57,82]. As mentioned above, this silver oxide phase is only present as a surface layer around the formed silver nanoparticles, which will be discussed further.

### 3.4. Faceting of the Dewetted Nanostructures and Micro-Cristallinity

**Figure 13:**
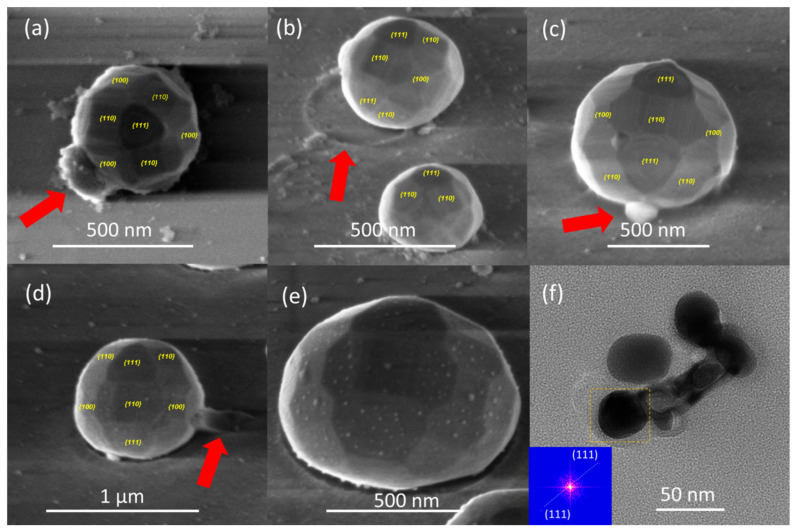
(**a**–**e**) Representative FE-SEM micrograph images of different Ag nanoparticles obtained by the solid-state thermal dewetting process. (**a**) top- and (**b**–**e**) tilted-view images to show the different low-index facets to highlight various features. (**f**) Representative HR-TEM micrograph image of silver nanoparticles obtained by the SSD process. Inset of (**f**) shows the FFT pattern extracted from the HR-TEM micrograph of the Ag NP enclosed by a yellow dashed rectangle in panel (**f**). Samples depicted in (**a**–**c**,**f**) have been obtained by applying the constant temperature region program depicted in Figure 1 with the following SSD process parameters: (**a**) d_Ag_ = 40 nm, T_A_ = 450 °C, t_A_ = 51 min; (**b**) d_Ag_ = 40 nm, T_A_ = 450 °C, t_A_ = 17 min; (**c**) d_Ag_ = 80 nm, T_A_ = 500 °C, t_A_ = 60 min; and (**f**) d_Ag_ = 80 nm, T_A_ = 500 °C, t_A_ = 17 min. The samples shown in (**d**,**e**) were obtained by applying the heat-up region program depicted in Figure 1 with the following SSD process parameters: d_Ag_ = 80 nm and T_A_ = 450 °C.

As a result of the SSD process, the formed Ag NPs particles show an almost spherical shape (planar size vertical size) and, additionally, equilibrium facets can be seen on the surface of them. In the synthesis and application of metallic nanostructures, crystallographic nanostructure faceting plays a crucial role [31,58]. The capacity to grow specific facets while eliminating others is largely the route by which nanostructures are shape-engineered [31,58]. Among the different SSD stages, the final one, in which irregularly shaped islands are reorganized into more favorable configurations, is essential in terms of determining nanostructure faceting [31,58]. In the case of freestanding metal nanostructures, the shape that minimizes the surface energy is commonly referred to as the Wulff shape [31,47,58,83]. However, when formed on a low-surface energy substrate, the nanostructure equilibrium shape still resembles the Wulff shape, but there, it is truncated by the substrate surface (for convenience, this will also be referred to as the Wulff shape) [31,47,58,83]. Figure 13a–d show high-magnification FE-SEM micrograph images of typical Ag NPs (formed under different SSD process parameters as indicated), which appear highly faceted and show a Wulff-like shape. It can be clearly seen that the Ag NPs exposed the {111} facets while facing up (see Figure 13a), with the Ag nanostructures orientated with their [111] axis perpendicular to the glass substrate surface (please see the procedure followed to assign the Miller indices to the exposed facets of the formed silver nanoparticles in SI). This is in agreement with the XRD results presented above, indicating out-of-plane [111] texturing. Furthermore, the Ag NPs exposed the co-presence of {111}, {110}, and {100} equilibrium facet characteristics (see Figure 13a–d), which can be attributed to a 26-facet rhombicuboctahedron crystal shape [84,85,86]. In fact, the most common equilibrium configuration for a face-centered-cubic (fcc) metal is a cuboctahedron with six (100) facets and eight (111) facets, which result from a surface energy hierarchy of γ_111_ < γ_100_ < γ_110_ [58,83,86].

Furthermore, the high-magnification FE-SEM micrograph images depicted in Figure 13a–e highlight features of both the SSD process leading to the formation of the Ag NPs and the formed dewettted Ag nanoparticles themselves. The first example is depicted in Figure 13a–d, in each micrograph image, the red arrow indicates a specific feature. During the SSD process, mass transfer from the connecting filament to the particles and until complete filament rupture occurs; in Figure 13a and in Figure 13c, the arrows indicate the residual signatures of the original retracted filament from which the particle originated. In addition, the red arrows in Figure 13b,d indicate the residual trace marks of the mass transfer on the glass substrate towards one of the Ag nanoparticles. Figure 13e shows a high-magnification FE-SEM micrograph image of a silver nanoparticle decorated by very tiny silver nanoparticles, which can be attributed to the last stages of the Ostwald ripening growth mechanism.

The finer details of the microstructure of the silver nanoparticles were further investigated by high resolution transmission electron microscopy (HR-TEM), which is illustrated in Figure 13f. The Inset of Figure 13f shows the fast Fourier transform (FFT) image of the marked zone of the Ag NP depicted in Figure 13f, demonstrating the high-quality single crystalline nature of this nanoparticle, which corresponds to cubic silver (111) crystallographic planes. 

### 3.5. Surface Chemical Analysis of the Ag Nanoparticle Arrays

Ag experiences oxidation when exposed to air. Jing et al. [87] studied the oxidation of Ag films when they are annealed in air and determined that films annealed at temperatures below 300 °C are not oxidized and that the Ag becomes oxidized at temperatures higher than 350 °C. The decomposition of the Ag oxide at 300 °C was studied by Herly and Prout [88]. The Ag oxide layer covering the Ag nanoparticles is formed as a surface passivation layer during the annealing process [38], AgO is expected to be less stable than Ag_2_O, and it decomposes according to the following reaction [89]:

(3) $$4\;\mathrm{AgO}\;⇋2\;{\mathrm{Ag}}_{2}O+O_{2}$$

**Figure 14:**
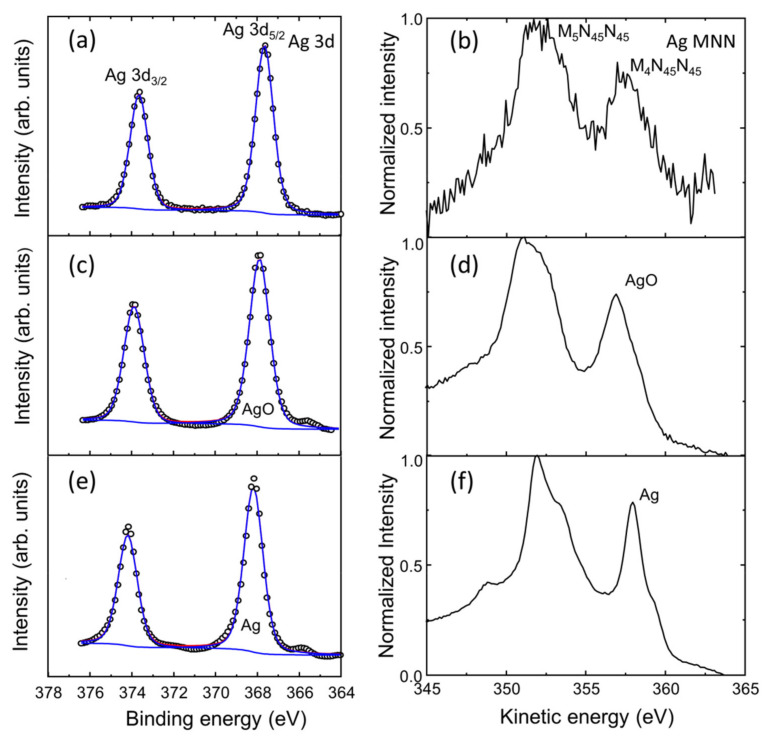
Evolution of high-resolution XPS Ag 3d and Ag MNN region spectra for a vacuum-thermal-evaporated silver layer with a nominal thickness d_Ag_ = 80 nm. (**a**,**b**) Surface study of the sample after the annealing step during the SSD process applying the constant temperature region program depicted in Figure 1 at T_A_ = 500 °C and for t_A_ = 26 min. Surface analysis of a silver layer with a nominal thickness d_Ag_ = 80 nm as-grown (without annealing): (**c**,**d**) and (**e**,**f**) before and after 1 min surface bombardment with Ar^+^ (~5 nm/min etching rate), respectively. Experimental data are presented by small open circles, while superimposed solid lines are the result of peak-fitting reconstruction.

As such in order to study the exact surface chemistry of the evolution of the as-grown silver layers before and after the formation of the Ag NPs through the SSD process, the X-ray photoelectron spectroscopy (XPS) surface analysis technique was employed. Figure 14 shows the evolution of high-resolution XPS Ag 3d and Ag MNN spectra regions for a vacuum-thermal-evaporated silver layer with a nominal thickness d_Ag_ = 80 nm for the as-grown silver layers and after the annealing step of the SSD process applying the constant temperature region program depicted in Figure 1 at T_A_ = 500 °C and for t_A_ = 26 min. The silver oxides show an anomalous negative binding energy shift compared to the metal with binding energies and Auger parameter values for Ag_2_O and AgO that are quite similar, making it quite difficult to determine the species of the oxides. For Ag_2_O and AgO the binding energy shifts from metallic Ag are approximately −0.4 eV and −0.8 eV [90]. The XPS Ag 3d region was deconvoluted for the Ag and Ag oxides contributions that were fixed to Ag 3d_5/2_ at the binding energies of 368.2 eV (full width at half maximum (FWHM) 1.12 eV), 368.3 eV (FWHM 1.20), and 367.8 eV (FWHM 1.25 eV) for Ag, Ag_2_O, and AgO, respectively [89,90,91]. A FWHM for AgO ranging from 1.2 to 1.6 has been proposed for Ag 3d_5/2_ [91,92]. The separation between Ag 3d_5/2_ and Ag 3d_3/2_ was 6 eV. The O1s region sample was not useful for determining the oxygen proportion of the oxide because of the surface contamination and the contribution of the substrate. However, the Auger parameters allowed us to confirm the presence of oxides in the outer layer of the Ag particles.

Through the above experiments we concluded that the silver layer obtained at the surface was AgO (Auger parameter of 724.8 eV) and metallic silver after 1 min of etching with Ar^+^ 4 kV (Auger parameter of 726.2 eV). Figure 14 shows also the XPS Ag MNN region; the two most intense peaks in the Auger structure the oxidized forms of the Ag and the reduced one, M_4_N_45_N_45,_ to be distinguished from each other based on the kinetic energy value of 357.9 eV for the reduced form and 356.9 eV for the oxidized form [90]. Both oxides show similar XPS MNN spectra patterns, and their patterns are well-differentiated from that of MNN for metallic silver [89]. The sample submitted to the SSD process for t_A_ = 26 min exhibited a deeper oxidized state (see Figure 14a,b). The amount of silver oxide in the outer surface layers of the Ag NPs increased with the annealing time, which was corroborated by the fact that the AgO phase was detected by XRD for a sample with an annealing time of 60 min (see Figure 12).

## 4. Conclusions

In this work, silver nanoparticle arrays on glass substrates were obtained by a solid-state thermal dewetting process for vacuum-evaporated polycrystalline silver precursor layers. The thermal treatment promoted the morphological modification of the films, leading to the Ag NPs having a discrete structure. The silver size, shape, surface density, and inter-nanoparticle distance of the nanoparticles was dependent on the initial film thickness, annealing temperature, and annealing time. Additionally, the shape of the NPs evolved from elongated at lower temperatures to circular as the temperature increased, and at longer annealing times, the circularity diminished slightly. The quasi-periodic nature of the Ag NPs arrays was quantified at length scales ranging from 0.9 to 2.8 micrometers. The Ag nanoparticles exhibited a cubic silver structure with (111) as the preferred crystallographic orientation. The presence of a monoclinic silver oxide phase was identified in samples annealed at the highest studied annealing temperatures and annealing times. Faceted Ag NPs were obtained with a 26-facet rhombicuboctahedron crystal shape, exposing the co-presence of the equilibrium facet characteristics of {111}, {110}, and {100}. Through XPS surface analysis, the presence AgO on the surface around the silver nanoparticles that was synthesized at the highest assayed temperature was detected. In conclusion, this work paves the way for the design and synthesis of silver nanoparticle arrays for further applications in engineering plasmonics in optical, electronic, and catalytic systems.

## Data Availability

Not applicable.

## Supplementary Materials

The following supporting information can be downloaded at: https://www.mdpi.com/article/10.3390/nano12040617/s1, Figure S1: FE-SEM micrographs and silver nanoparticle size distribution histograms for vacuum-thermal-evaporated silver layers with a nominal thickness d_Ag_ = 40 nm at different stages of the annealing step of the SSD process applying the heat-up region program depicted in Figure 1 and ending at different T_A_ values as indicated. Figure S2: FE-SEM micrographs and silver nanoparticle size distribution histograms for vacuum-thermal-evaporated silver layers with a nominal thickness d_Ag_ = 80 nm at different stages of the annealing step of the SSD process applying the heat-up region program depicted in Figure 1 and ending at different T_A_ values as indicated. Figure S3: Typical normalized measured radially averaged autocorrelation function, g(r), for the silver nanoparticle positions for typical samples with d_Ag_ = 40 nm and different T_A_ values, as indicated. Figure S4: Typical normalized and radially measured averaged autocorrelation function, g(r), for the silver nanoparticle positions for typical samples with d_Ag_ = 80 nm and different T_A_ values, as indicated. Figure S5: Top view of FE-SEM micrographs t and silver nanoparticle size distribution histograms of vacuum-thermal-evaporated silver layers with a nominal thickness d_Ag_ = 40 nm at different stages of the annealing step of the SSD process by applying the constant temperature region program depicted in Figure 1 at T_A_ = 450 °C and ending at different t_A_ values, as indicated. Figure S6: Top views of FE-SEM micrographs and silver nanoparticle size distribution histograms of vacuum-thermal-evaporated silver layers with a nominal thickness d_Ag_ = 80 nm at different stages of the annealing step of the SSD process by applying the constant temperature region program depicted in Figure 1 at T_A_ = 500 °C and ending at different t_A_ values, as indicated. Figure S7: Plot of silver nanoparticle average size (D) square as a function of the annealing time t_A_ of the annealing step of the SSD process. Figure S8: FE-SEM micrograph images of vacuum-thermal-evaporated silver layers with four nominal thickness values: (a) d_Ag_ = 20 nm, (b) 40 nm, (c) 60 nm, and (d) 80 nm annealed at T_A_ = 400 °C (applying the temperature-time plateau region program depicted in Figure 1) with t_A_ = 1 h. Structural parameter definitions are also given: the texture coefficient TC*(hkl)* and the Scherrer equation applied to the calculation is the crystallite coherent scattering domain. The procedure used to determine surface crystallography of the faceted silver nanoparticles is also provided.
