# Supplementary material for: Silver Nanoparticle Arrays onto Glass Substrates Obtained by Solid-State Thermal Dewetting: A Morphological, Structural and Surface Chemical Study
Source: Nanomaterials (Basel). 2022 Feb 11;12(4):617. doi: 10.3390/nano12040617 (PMC8878931; doi:10.3390/nano12040617)
Supplement: Supplementary file 1 [file nanomaterials-12-00617-s001.zip › nanomaterials-1585429 SI.pdf]

SUPPORTING INFORMATION

Silver Nanoparticle Arrays onto Glass Substrates Obtained by Solid-State Thermal Dewetting: A Morphological, Structural and Surface Chemical Study

Juan Agustín Badán[1], Elena Navarrete-Astorga[2], Rodrigo Henríquez[3], Francisco Martín Jiménez [2], Daniel Ariosa[1], José Ramón Ramos-Barrado[2] and Enrique A. Dalchiele[1,*].

[1] Instituto de Física, Facultad de Ingeniería, Universidad de la República, Julio Herrera y Reissig 565, C.C. 30, 11000 Montevideo, Uruguay; abadan@fing.edu.uy; dariosa@fing.edu.uy; dalchiel@fing.edu.uy

[2] Laboratorio de Materiales y Superficies (Unidad Asociada al CSIC). Departamentos de Física Aplicada & Ing. Química, Universidad de Málaga, E29071 Málaga, Spain; enavarrete@uma.es; marjim@uma.es; barrado@uma.es

[3] Instituto de Química, Facultad de Ciencias, Pontificia Universidad Católica de Valparaíso, Av. Brasil, 2950, Valparaíso, Chile; rodrigo.henriquez@pucv.cl

* Correspondence: dalchiel@fing.edu.uy ; Tel.: (+598 27142714)

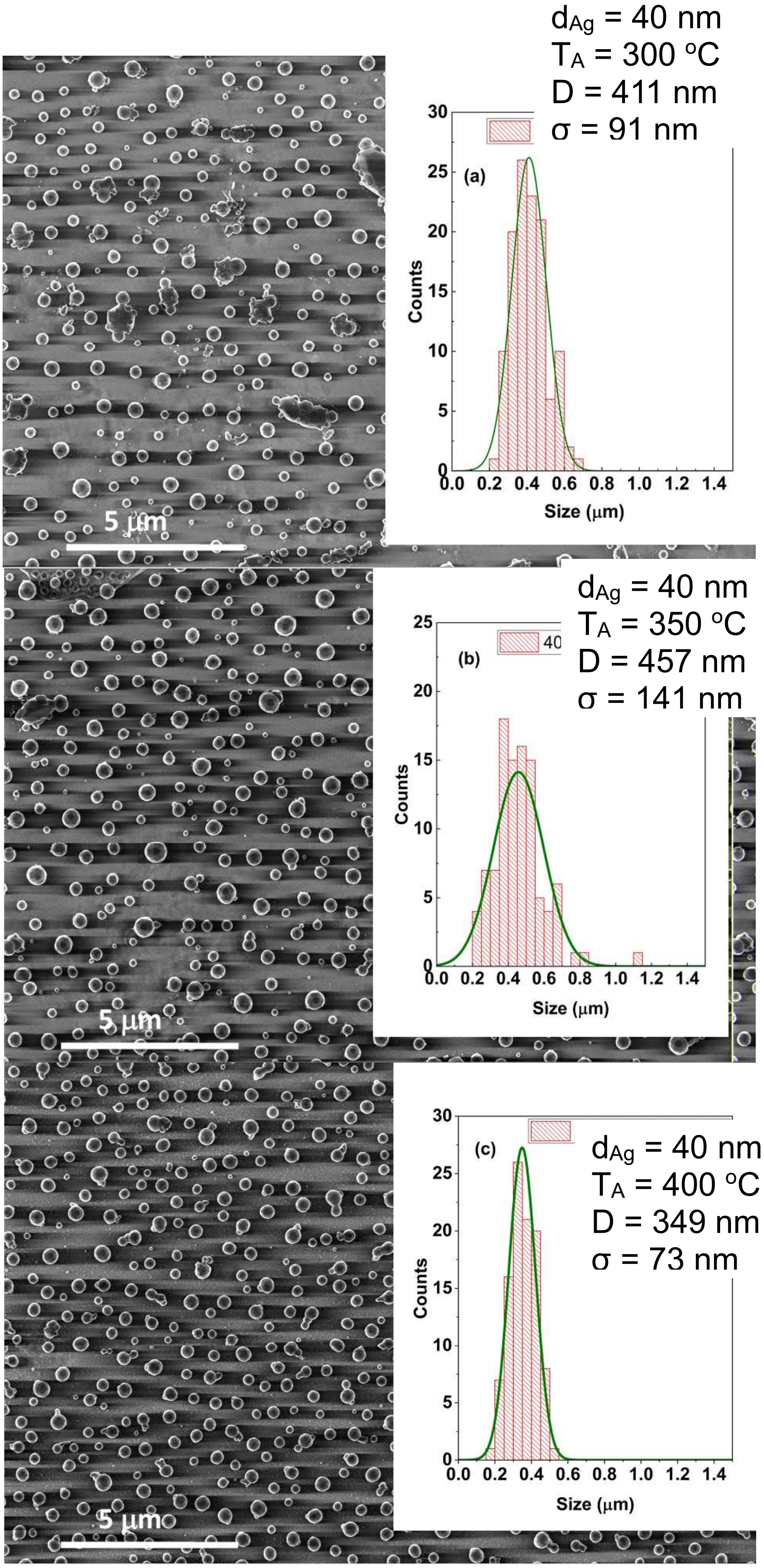
dAg = 40 nm
TA = 300 oC
D = 411 nm
σ = 91 nm
(a)
Counts
Size (μm)
5 μm
dAg = 40 nm
TA = 350 oC
D = 457 nm
σ = 141 nm
(b)
Counts
Size (μm)
5 μm
dAg = 40 nm
TA = 400 oC
D = 349 nm
σ = 73 nm
(c)
Counts
Size (μm)
5 μm

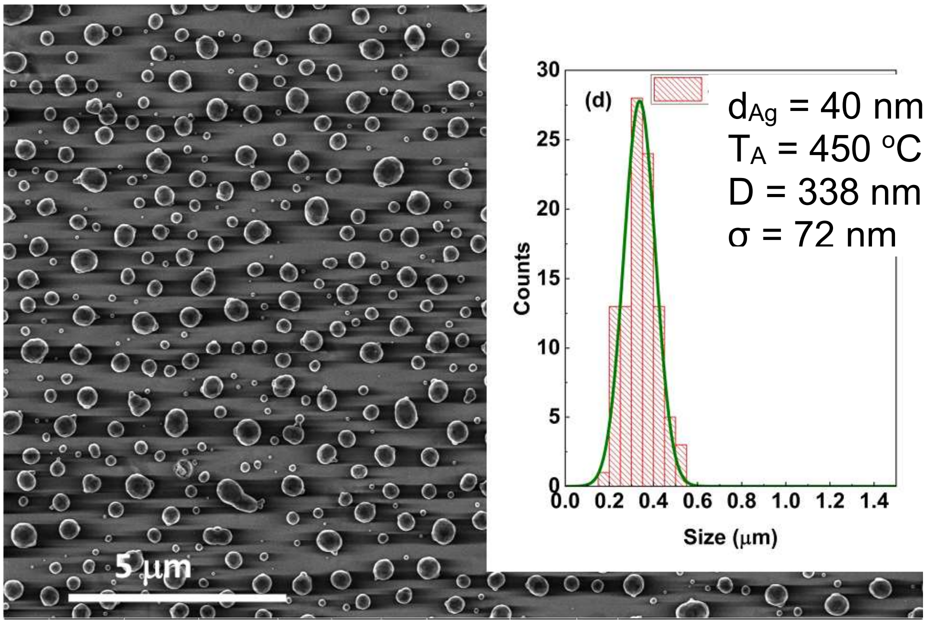

Figure S1. FE-SEM micrographs and silver nanoparticle size distribution histograms for vacuum thermal evaporated silver layers with a nominal thickness d_{Ag} = 40 nm at different stages of the annealing step of the SSD process by applying the heat-up region program depicted in Figure 1 and ending at different T_A values as indicated. The mean size D of the formed silver nanoparticles and standard deviation σ of the size distribution are indicated.

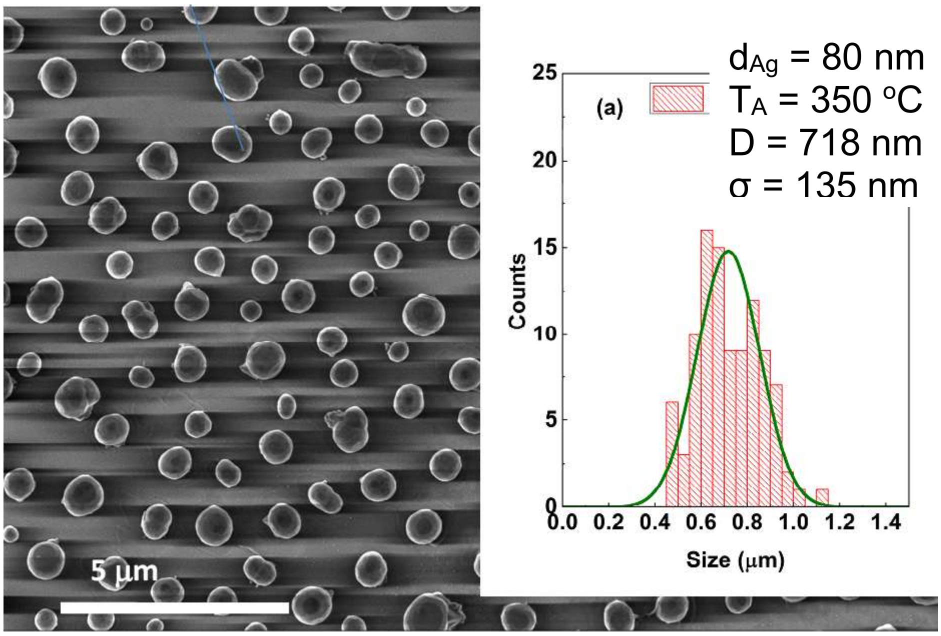

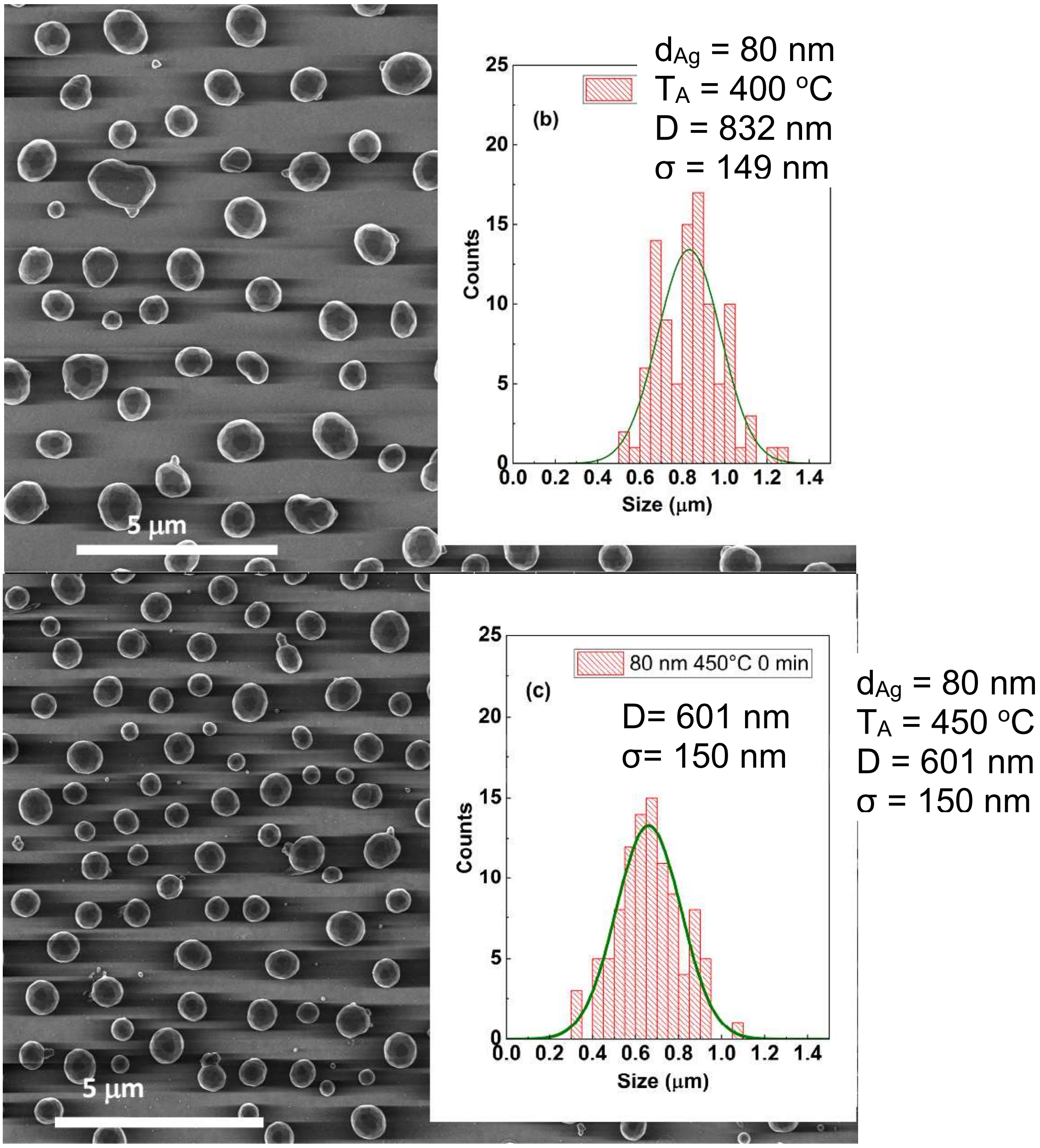
5 μm
(b)
d_{Ag} = 80 nm
T_A = 400 °C
D = 832 nm
σ = 149 nm
Counts
Size (μm)
5 μm
(c)
80 nm 450°C 0 min
D= 601 nm
σ= 150 nm
Counts
Size (μm)
d_{Ag} = 80 nm
T_A = 450 °C
D = 601 nm
σ = 150 nm

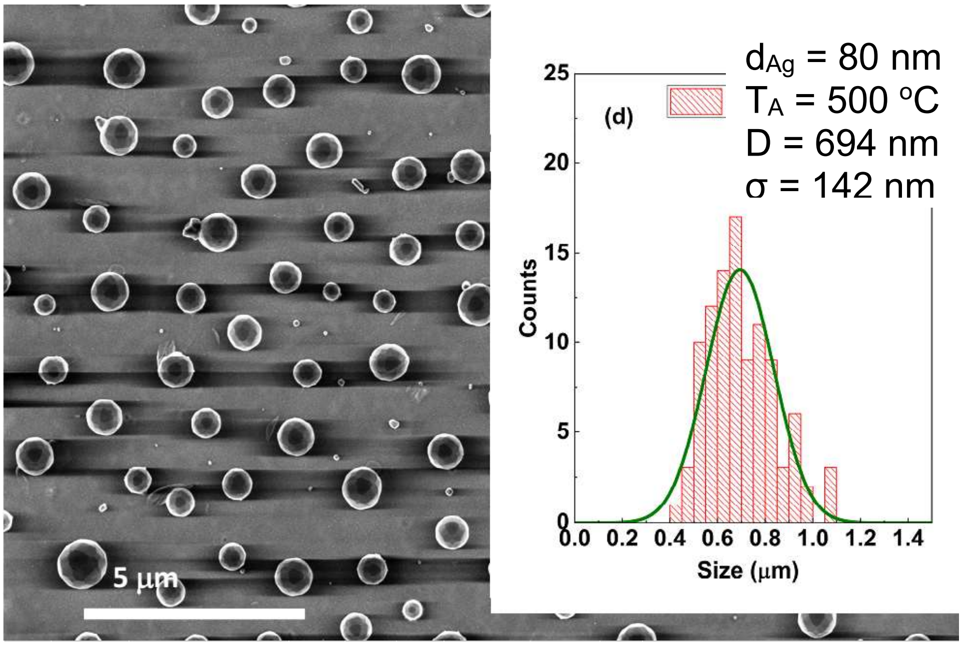

Figure S2. FE-SEM micrographs and silver nanoparticle size distribution histograms for vacuum thermal evaporated silver layers with a nominal thickness d_{Ag} = 80 nm at different stages of the annealing step of the SSD process by applying the heat-up region program depicted in Figure 1 and ending at different T_A values as indicated. The mean size D of the formed silver nanoparticles and standard deviation σ of the size distribution are indicated.

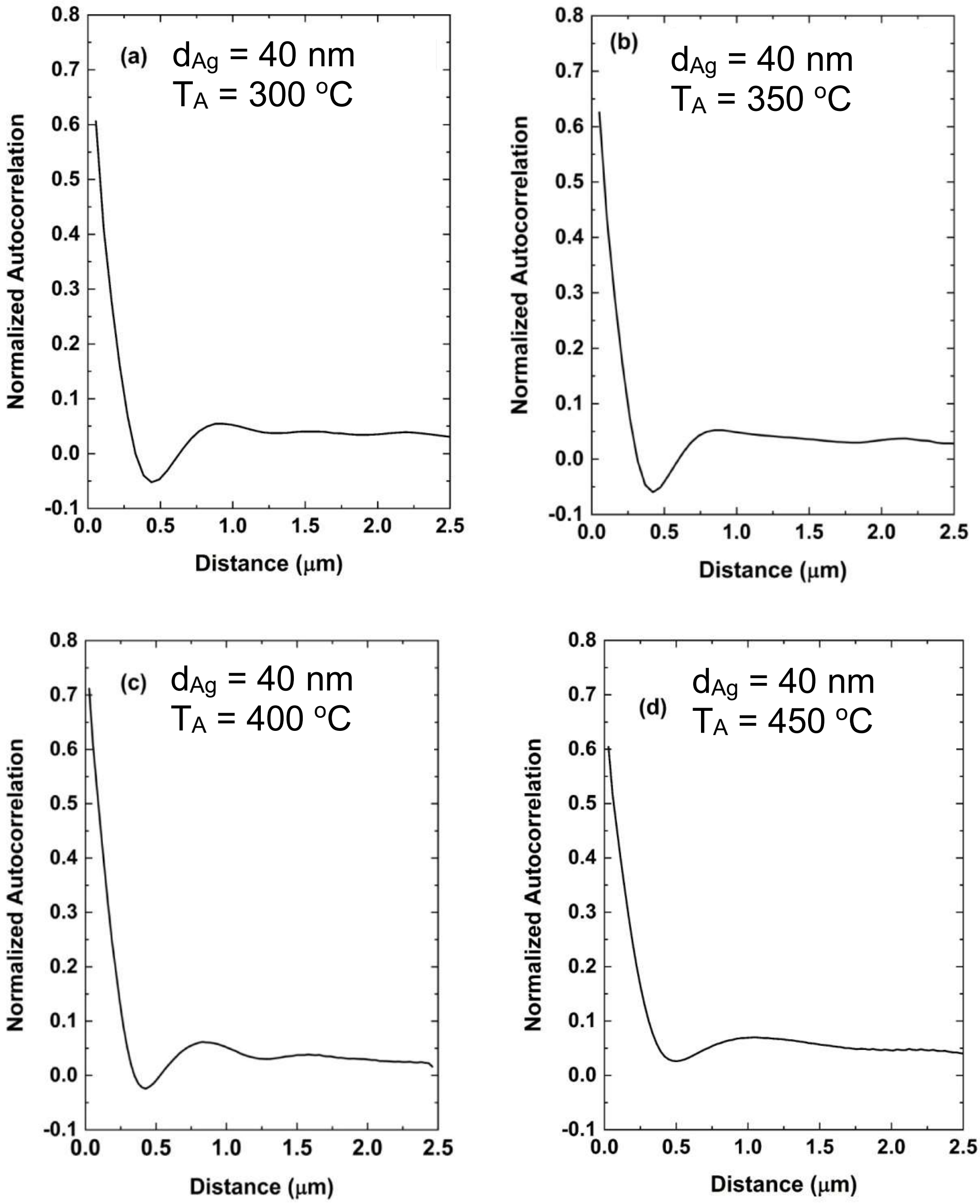

Figure S3. Typical normalized measured radially averaged autocorrelation function, g(r), for the silver nanoparticle positions for typical samples with dAg = 40 nm and different TA values as indicated.

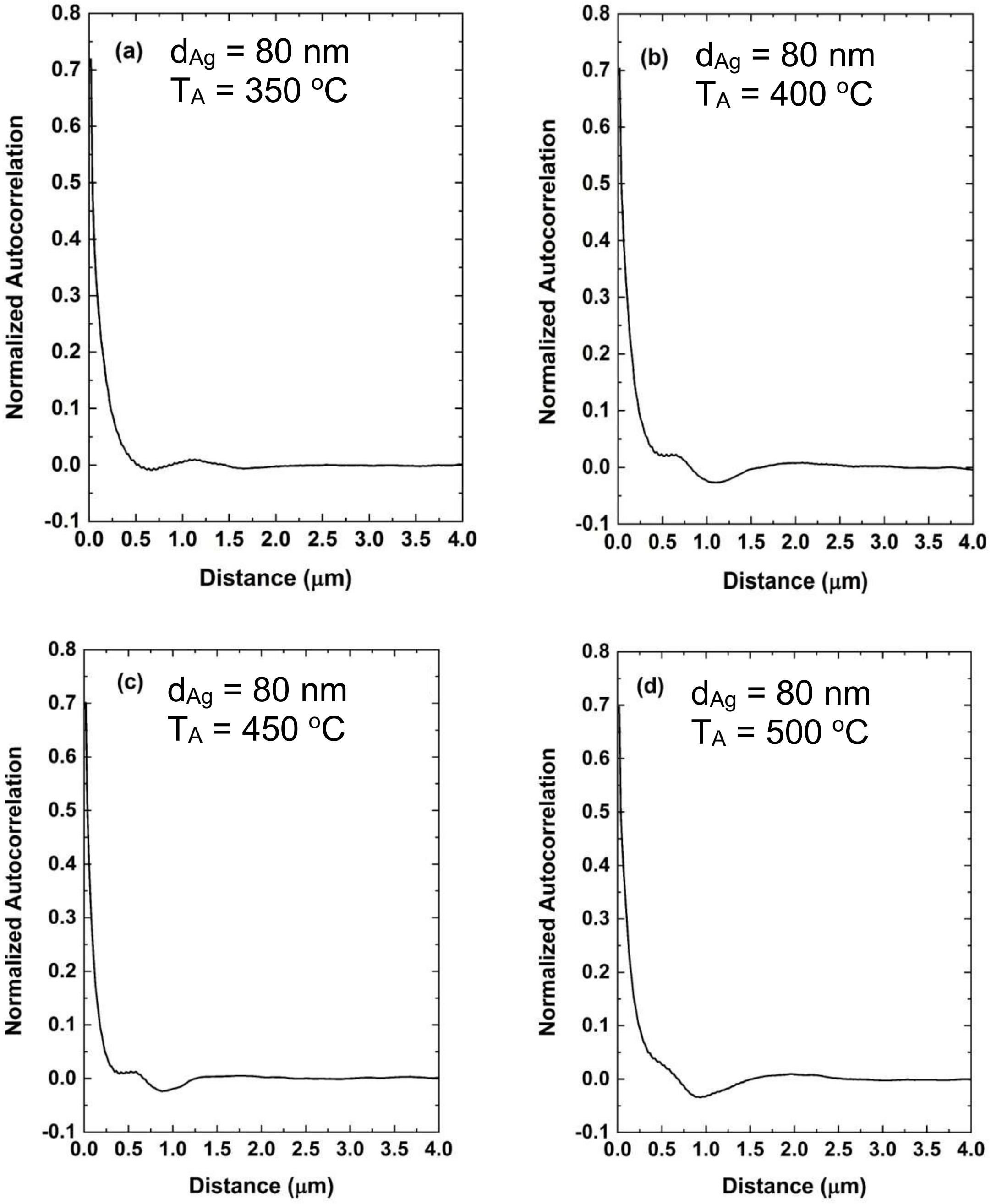

Figure S4. Typical normalized measured radially averaged autocorrelation function, g(r), for the silver nanoparticle positions for typical samples with dAg = 80 nm and different TA values as indicated.

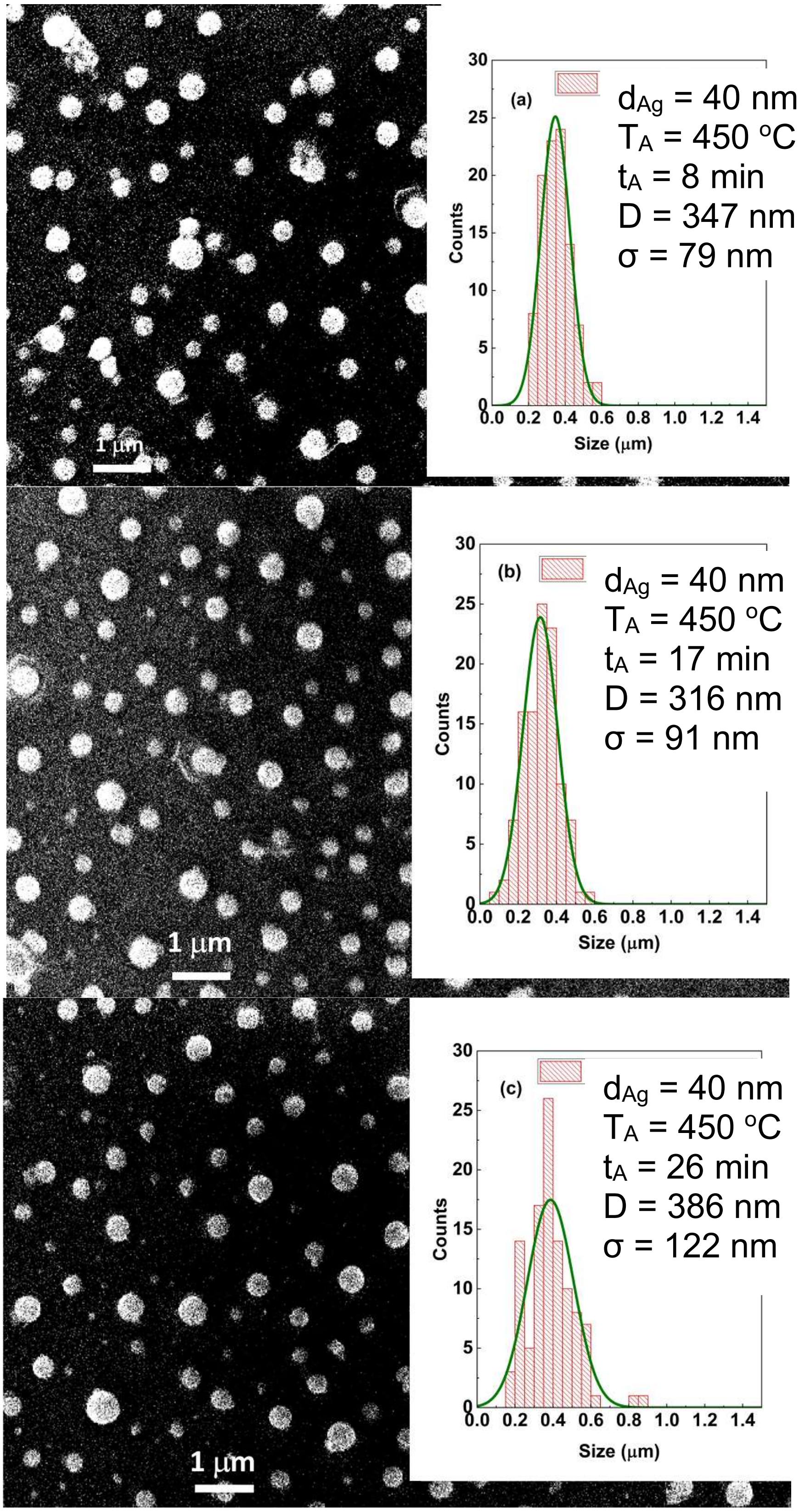
(a)
dAg = 40 nm
TA = 450 oC
tA = 8 min
D = 347 nm
σ = 79 nm
Counts
Size (μm)
1 μm
(b)
dAg = 40 nm
TA = 450 oC
tA = 17 min
D = 316 nm
σ = 91 nm
Counts
Size (μm)
1 μm
(c)
dAg = 40 nm
TA = 450 oC
tA = 26 min
D = 386 nm
σ = 122 nm
Counts
Size (μm)
1 μm

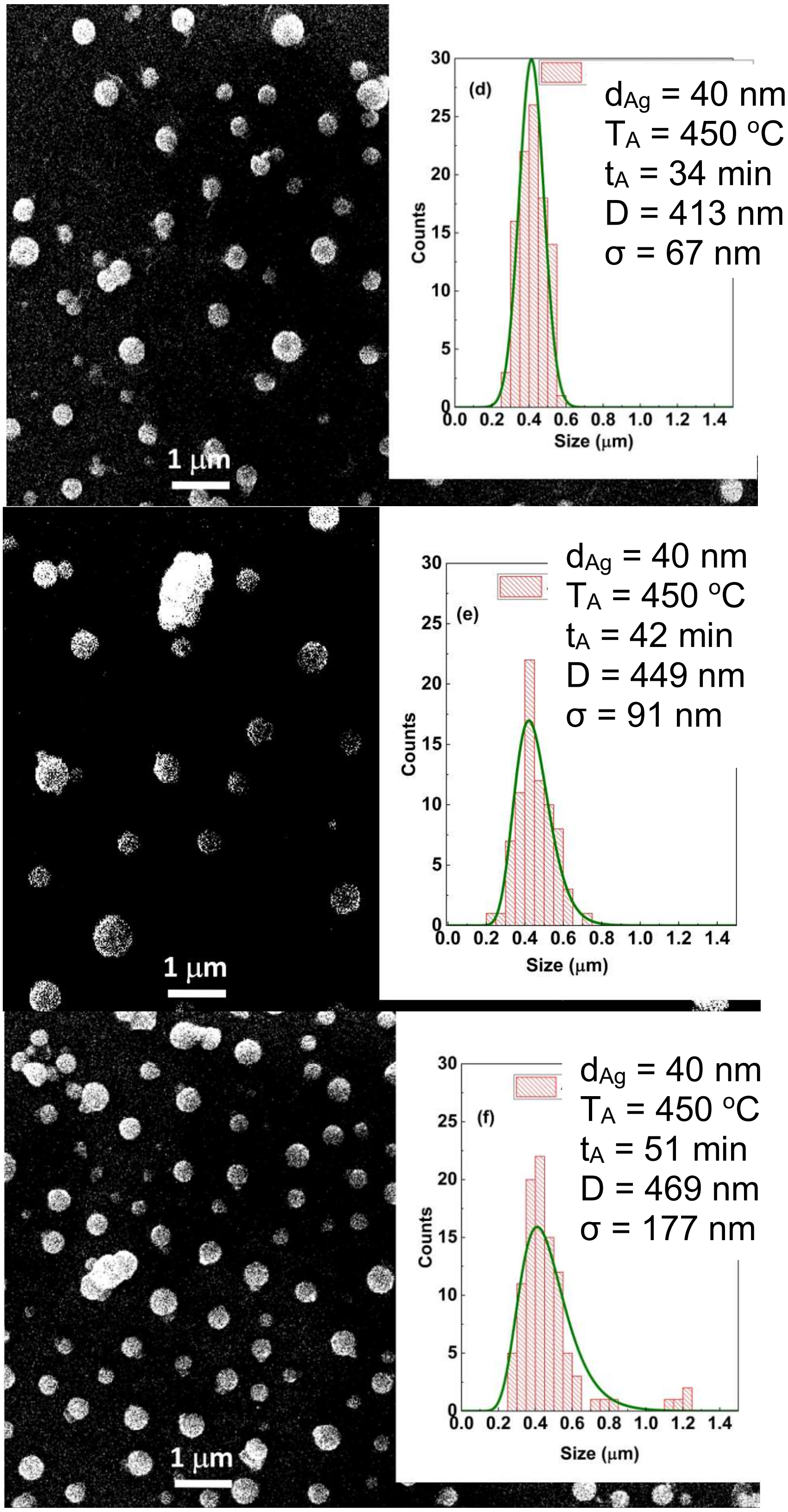

Figure S5. FE-SEM micrograph top views and silver nanoparticle size distribution histograms of vacuum thermal evaporated silver layers with a nominal thickness d_{Ag} = 40 nm at different stages of the annealing step of the SSD process by applying the constant temperature region program depicted in Figure 1 at T_A = 450 °C and ending at different t_A values as indicated. The mean size D of the formed silver nanoparticles and standard deviation σ of the size distribution are indicated.

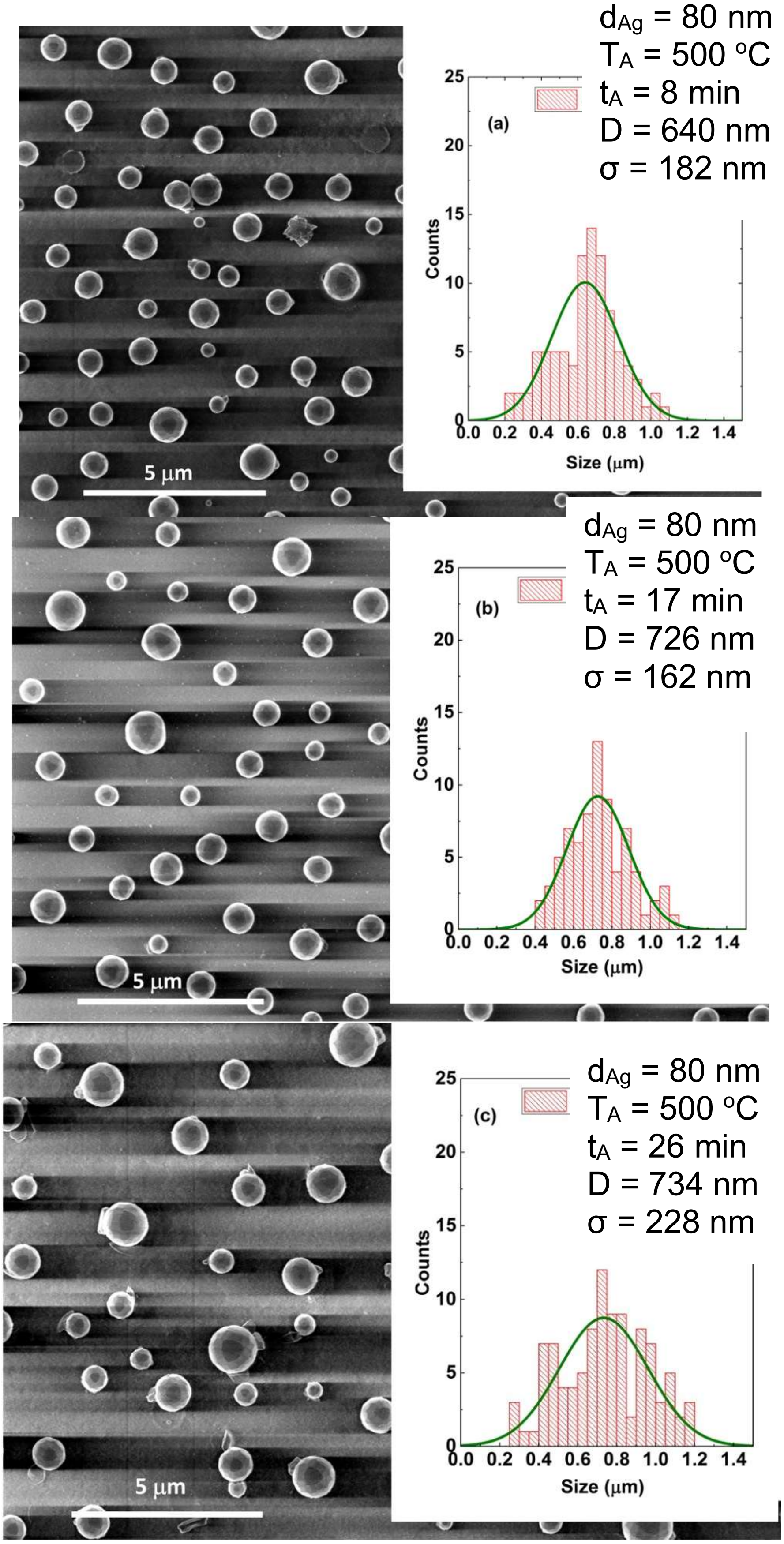

dAg = 80 nm
TA = 500 oC
tA = 8 min
D = 640 nm
σ = 182 nm
(a)
Counts
Size (μm)
5 μm
dAg = 80 nm
TA = 500 oC
tA = 17 min
D = 726 nm
σ = 162 nm
(b)
Counts
Size (μm)
5 μm
dAg = 80 nm
TA = 500 oC
tA = 26 min
D = 734 nm
σ = 228 nm
(c)
Counts
Size (μm)
5 μm

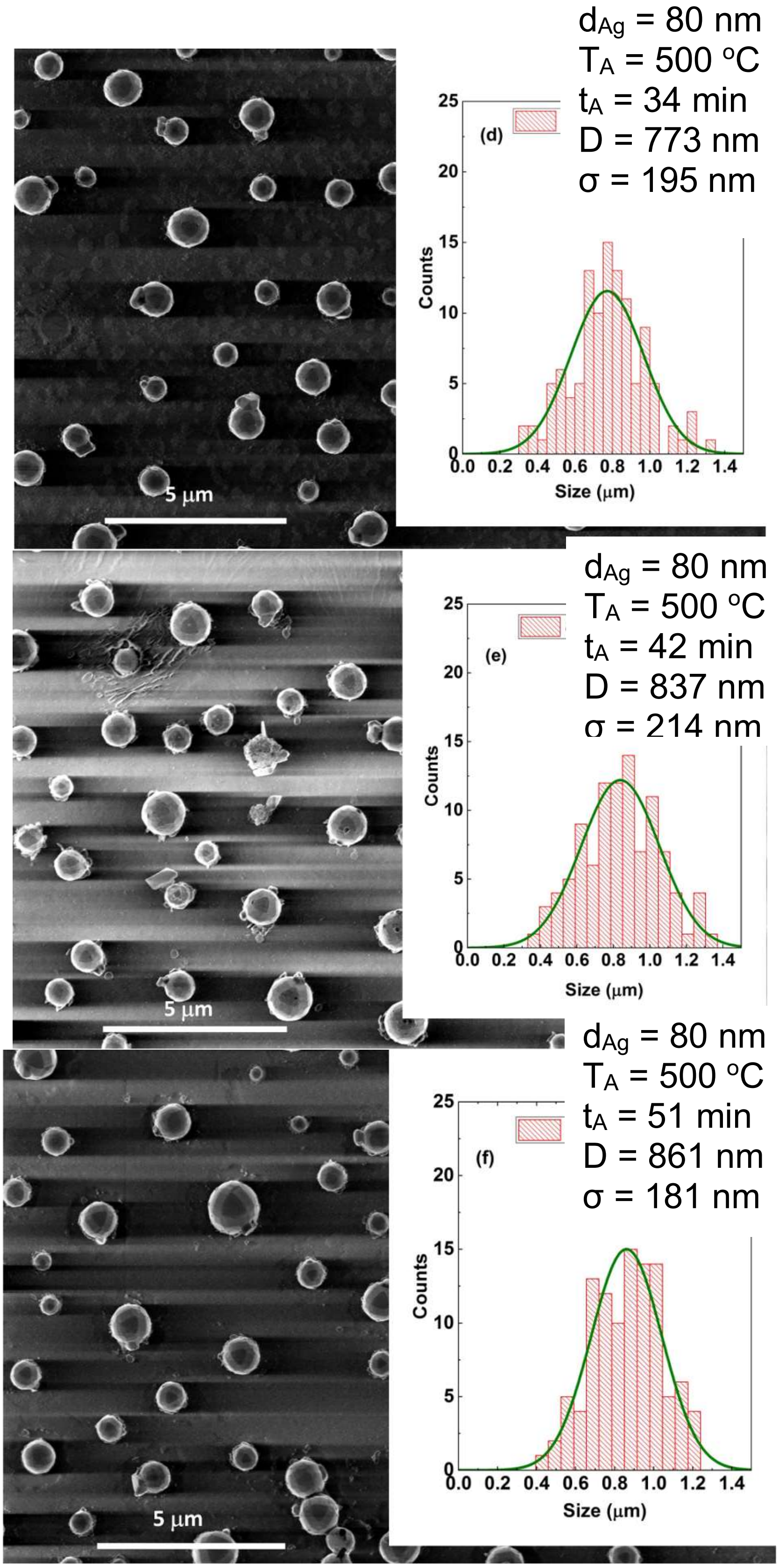

Figure S6. FE-SEM micrograph top views and silver nanoparticle size distribution histograms of vacuum thermal evaporated silver layers with a nominal thickness d_{Ag}=80 nm at different stages of the annealing step of the SSD process by applying the constant temperature region program depicted in Figure 1 at

T_A=500ºC and ending at different t_A values as indicated. The mean size D of the formed silver nanoparticles and standard deviation σ of the size distribution are indicated.

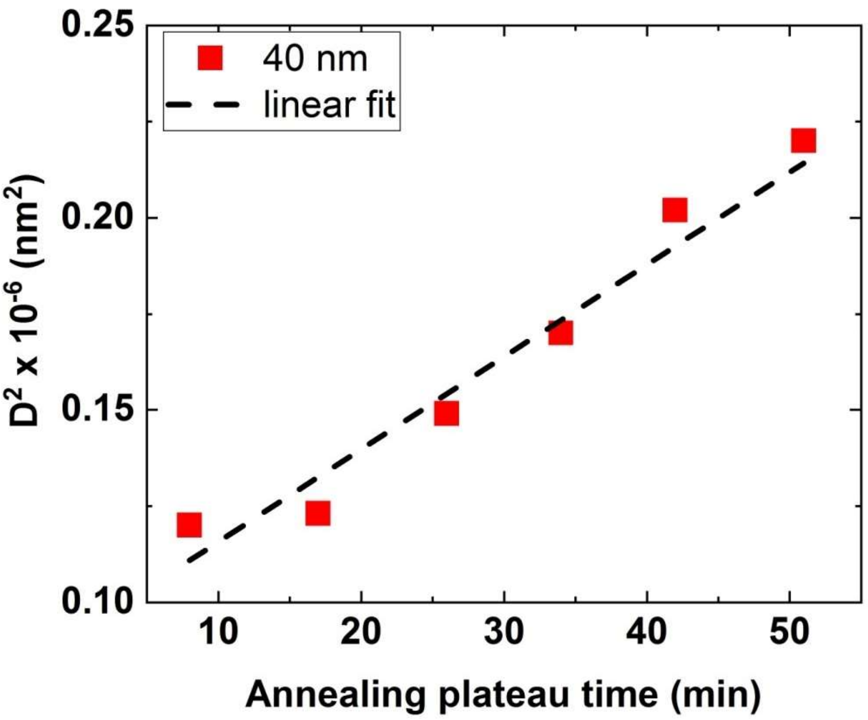

Figure S7. Plot of silver nanoparticle average size (D) square as a function of the annealing time t_A of the annealing step of the SSD process. The dashed black line corresponds to the linear fitting. SSD process for silver vacuum thermal evaporated layers with a nominal thickness d_{Ag}=40 nm by applying the constant temperature region program depicted in Fig. 1 at T_A=450 ºC.

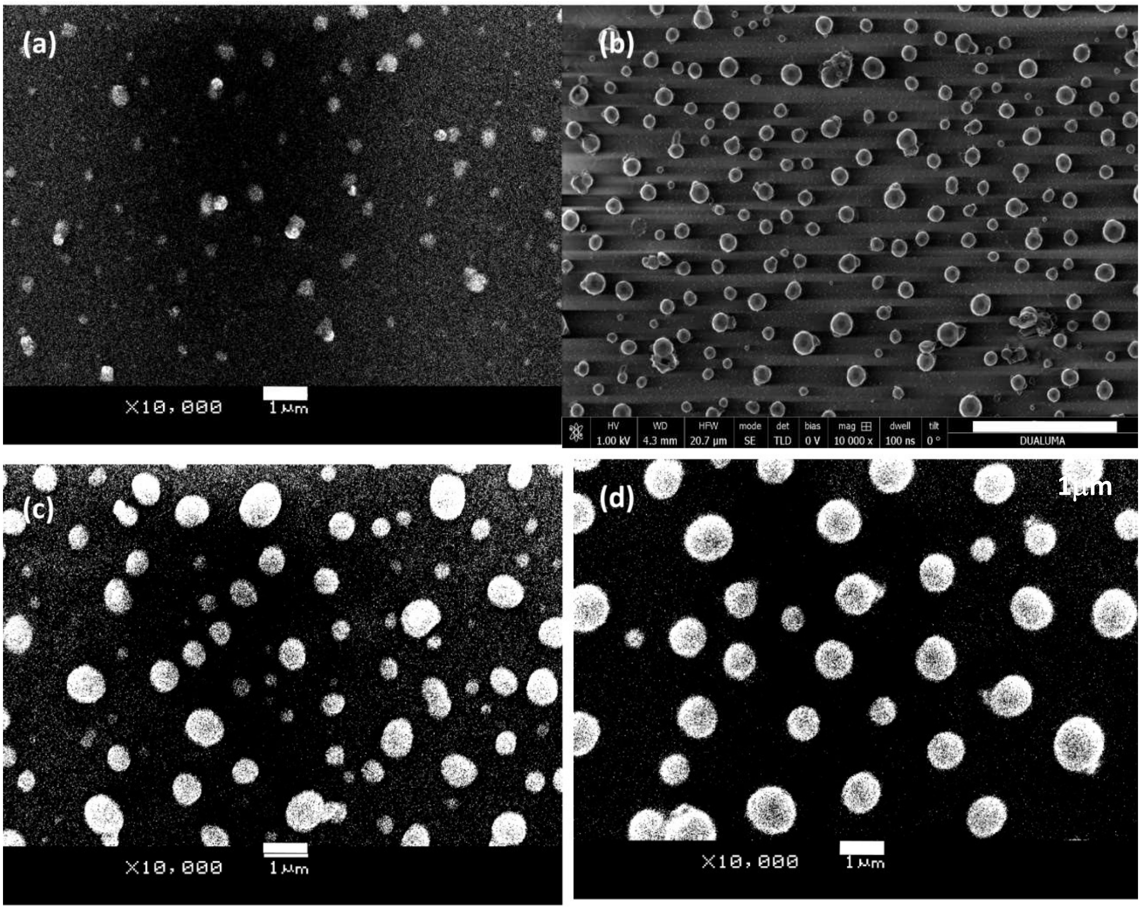

Figure S8. FE-SEM micrograph images of vacuum thermal evaporated silver layers with four nominal thickness values: (**a**) d_{Ag}=20 nm, (**b**) 40 nm, (**c**) 60 nm and (**d**) 80 nm annealed at T_A= 400 °C (by applying the temperature-time plateau region program depicted in Figure 1), with t_A= 1 hour. The white bars of a),c) and d) correspond to 1 m, whereas the one of b) corresponds to 5 m.

Structural parameters definition.

The texture coefficient TC(*hkl*) is calculated by means of the following equation [1–6]:

$$TC(hkl) = \frac{I_{(hkl)}/I_{0(hkl)}}{\left(\frac{1}{N}\right)\sum_N I_{(hkl)}/I_{0(hkl)}} \quad (1)$$

where$I_{(hkl)}$ and $I_{0(hkl)}$ are the intensity of the (*hkl*) Bragg reflection for the studied sample and for a randomly oriented sample, respectively, and N is the number of considered diffraction peaks. Basically, for randomly oriented grain samples, the texture coefficient equals 1. On the other hand, for perfectly orientated grain samples along the (hkl) direction, the texture coefficient equals N (for (*hkl*) planes) or 0 (for other crystallographic planes)[1–6]. In present case, only the three main cubic Ag diffraction peaks were considered (N=3), namely (111), (200) and (222).

The Scherrer equation is presented as follows[6–8]:

$$D_C = \frac{0{,}95\,\lambda}{\beta_{hk}\ \cos\theta_{hkl}} \quad (2)$$

where D_C is the crystallite coherently scattering domain calculated from (*hkl*) diffraction peak, λ is the wavelength used (1.54 Å), β_{hkl} is the corrected angular line width at half-maximum intensity in radians, and θ_{hkl} is Bragg's angle. The β_{hkl} parameter was corrected using the following equation:

$$\beta_{hkl} = \sqrt{\beta_{exp}^2 - \beta_{ins}^2} \quad (3)$$

where β_{exp} corresponds to the experimentally determined full width at half of the maximum intensity, FWHM, of the peak. The instrumental width was determined as β_{ins} = 0.14º by using a silicon standard pattern.

Determination of surface crystallography of the faceted silver nanoparticles.

The Miller indices of the exposed facets of the silver nanoparticles have been assigned in the basis of the geometrical shapes exhibited by each of these facets and the angle between them (according to Steno's law), and a further comparison to previously published works dealing with the same geometrical model of an ideal 26-facet rhombicuboctahedron microstructure, such as the paper of Liang et al. [9].

References

1. G.B. Harris, X. Quantitative measurement of preferred orientation in rolled uranium bars, London, Edinburgh, Dublin Philos. Mag. J. Sci. 43 (1952) 113–123. https://doi.org/10.1080/14786440108520972.
2. H.R. Moutinho, F.S. Hasoon, F. Abulfotuh, L.L. Kazmerski, Investigation of polycrystalline CdTe thin films deposited by physical vapor deposition, close-spaced sublimation, and sputtering, J. Vac. Sci. Technol. A. 13 (1995) 2877–2883. https://doi.org/10.1116/1.579607.
3. V. Consonni, G. Rey, H. Roussel, B. Doisneau, E. Blanquet, D. Bellet, Preferential orientation of fluorine-doped SnO2 thin films: The effects of growth temperature, Acta Mater. 61 (2013) 22–31. https://doi.org/https://doi.org/10.1016/j.actamat.2012.09.006.
4. E. A. Dalchiele, P. Giorgi, R.E. Marotti, F. Mart í In, J.R. Ramos-Barrado, R. Ayouci, D. Leinen, Electrodeposition of ZnO thin films on n-Si(1 0 0), Sol. Energy Mater. Sol. Cells. 70 (2001) 245–254. https://doi.org/10.1002/anie.201001827.
5. H.R. Moutinho, M.M. Al-Jassim, D.H. Levi, P.C. Dippo, L.L. Kazmerski, Effects of CdCl2 treatment on the recrystallization and electro-optical properties of CdTe thin films, J. Vac. Sci. Technol. A. 16 (1998) 1251–1257. https://doi.org/10.1116/1.581269.
6. G. Riveros, D. Ramírez, D.L. Gau, L. Hernández, P. Häberle, R.E. Marotti, R. Romero, A. Cuevas, F. Martín, E.A. Dalchiele, Electrodeposition of Single Phase SnS Thin Films: Effect of Electrolytic Bath Temperature on the Final Film Properties, J. Electrochem. Soc. 166 (2019) D44–D51. https://doi.org/10.1149/2.0661902jes.

7. P. Scherrer, Nachrichten von der Gesellschaft der Wissenschaften zu Göttingen, Math. Klasse. 2 (1918) 98–100.
8. A.L. Patterson, The Scherrer Formula for X-Ray Particle Size Determination, Phys. Rev. 56 (1939) 978–982. https://doi.org/10.1103/PhysRev.56.978.
9. C. Liang, S. Huang, W. Zhao, W. Liu, J. Chen, H. Liu and Y. Tong, Polyhedral Fe_3O_4 nanoparticles for lithium ion storage, *New J. Chem.*, 39 (2015) 2651-2656.
